# SARS-CoV-2 Viral Load in the Nasopharynx at Time of First Infection Among Unvaccinated Individuals: A Secondary Cross-Protocol Analysis of 4 Randomized Trials

**DOI:** 10.1001/jamanetworkopen.2024.12835

**Published:** 2024-05-23

**Authors:** Leigh H. Fisher, Jia Jin Kee, Albert Liu, Claudia M. Espinosa, April K. Randhawa, James Ludwig, Craig A. Magaret, Samuel T. Robinson, Peter B. Gilbert, Ollivier Hyrien, James G. Kublin, Nadine Rouphael, Ann R. Falsey, Magdalena E. Sobieszczyk, Hana M. El Sahly, Beatriz Grinsztejn, Glenda E. Gray, Karen L. Kotloff, Cynthia L. Gay, Brett Leav, Ian Hirsch, Frank Struyf, Lisa M. Dunkle, Kathleen M. Neuzil, Lawrence Corey, Yunda Huang, Paul A. Goepfert, Stephen R. Walsh, Lindsey R. Baden, Holly Janes

**Affiliations:** 1 Vaccine and Infectious Disease Division, Fred Hutchinson Cancer Center, Seattle, Washington; 2 Bridge HIV, San Francisco Department of Public Health, San Francisco, California; 3 University of South Florida Morsani College of Medicine, Tampa; 4 Hope Clinic, Emory University, Atlanta, Georgia; 5 Infectious Disease Division, University of Rochester, Rochester, New York; 6 Department of Medicine, Columbia University Irving Medical Center, New York, New York; 7 Department of Molecular Virology and Microbiology, Baylor College of Medicine, Houston, Texas; 8 Evandro Chagas National Institute of Infectious Diseases-Oswaldo Cruz Foundation, Rio de Janeiro, Brazil; 9 South African Medical Research Council, Cape Town, South Africa; 10 Center for Vaccine Development and Global Health, Department of Pediatrics, University of Maryland School of Medicine, Baltimore; 11 University of North Carolina School of Medicine, Chapel Hill; 12 Moderna Inc, Cambridge, Massachusetts; 13 Vaccines & Immune Therapies, BioPharmaceuticals R&D, AstraZeneca, Cambridge, United Kingdom; 14 Janssen Research and Development, Beerse, Belgium; 15 Novavax Inc, Gaithersburg, Maryland; 16 Center for Vaccine Development and Global Health, University of Maryland School of Medicine, Baltimore, Maryland; 17 University of Alabama at Birmingham Heersink School of Medicine, Birmingham; 18 Brigham & Women’s Hospital, Boston, Massachusetts

## Abstract

**Importance:**

SARS-CoV-2 viral load (VL) in the nasopharynx is difficult to quantify and standardize across settings, but it may inform transmission potential and disease severity.

**Objective:**

To characterize VL at COVID-19 diagnosis among previously uninfected and unvaccinated individuals by evaluating the association of demographic and clinical characteristics, viral variant, and trial with VL, as well as the ability of VL to predict severe disease.

**Design, Setting, and Participants:**

This secondary cross-protocol analysis used individual-level data from placebo recipients from 4 harmonized, phase 3 COVID-19 vaccine efficacy trials sponsored by Moderna, AstraZeneca, Janssen, and Novavax. Participants were SARS-CoV-2 negative at baseline and acquired COVID-19 during the blinded phase of the trials. The setting included the US, Brazil, South Africa, Colombia, Argentina, Peru, Chile, and Mexico; start dates were July 27, 2020, to December 27, 2020; data cutoff dates were March 26, 2021, to July 30, 2021. Statistical analysis was performed from November 2022 to June 2023.

**Main Outcomes and Measures:**

Linear regression was used to assess the association of demographic and clinical characteristics, viral variant, and trial with polymerase chain reaction–measured log_10_ VL in nasal and/or nasopharyngeal swabs taken at the time of COVID-19 diagnosis.

**Results:**

Among 1667 participants studied (886 [53.1%] male; 995 [59.7%] enrolled in the US; mean [SD] age, 46.7 [14.7] years; 204 [12.2%] aged 65 years or older; 196 [11.8%] American Indian or Alaska Native, 150 [9%] Black or African American, 1112 [66.7%] White; 762 [45.7%] Hispanic or Latino), median (IQR) log_10_ VL at diagnosis was 6.18 (4.66-7.12) log_10_ copies/mL. Participant characteristics and viral variant explained only 5.9% of the variability in VL. The independent factor with the highest observed differences was trial: Janssen participants had 0.54 log_10_ copies/mL lower mean VL vs Moderna participants (95% CI, 0.20 to 0.87 log_10_ copies/mL lower). In the Janssen study, which captured the largest number of COVID-19 events and variants and used the most intensive post-COVID surveillance, neither VL at diagnosis nor averaged over days 1 to 28 post diagnosis was associated with COVID-19 severity.

**Conclusions and Relevance:**

In this study of placebo recipients from 4 randomized phase 3 trials, high variability was observed in SARS-CoV-2 VL at the time of COVID-19 diagnosis, and only a fraction was explained by individual participant characteristics or viral variant. These results suggest challenges for future studies of interventions seeking to influence VL and elevates the importance of standardized methods for specimen collection and viral load quantitation.

## Key Points

**Question:**

What factors are associated with SARS-CoV-2 viral load at the time of COVID-19 diagnosis, and is viral load associated with disease severity?

**Findings:**

In this secondary cross-protocol analysis of 1667 placebo recipients from 4 harmonized, randomized, phase 3 COVID-19 vaccine efficacy trials, no associations were found between viral load and any of the measured covariates or disease severity.

**Meaning:**

The findings of this study suggest that caution should be exercised in the use of individual-level viral load in comparisons across trials and/or settings and as a surrogate for COVID-19 severity, especially given increasing diversity in preexisting immunity.

## Teaser

This secondary cross-protocol analysis investigates the viral load at COVID-19 diagnosis among previously uninfected and unvaccinated individuals by demographic and clinical characteristics, viral variant, and trial, as well as the ability of viral load to predict severe disease.

## Introduction

There have been more than 750 million confirmed SARS-CoV-2 infections worldwide since the start of the COVID-19 pandemic.^1^ Understanding drivers of transmission is critical for addressing issues of public health, developing outbreak mitigation policies, and informing individual decision-making. Numerous lines of evidence point to viral load (VL) as a marker of transmission potential.^2,3,4,5,6,7^ SARS-CoV-2 VL at or near the time of hospitalization has been associated with symptom severity and mortality^8,9,10,11,12^ and used as an end point in COVID-19 treatment trials.^13,14,15,16,17,18,19^ However, accurately measuring VL is challenging: it is dynamic, typically peaking before or soon after onset of symptoms; it is highly variable across participants; it differs by specimen type and adequacy of collection; and results from different assays and laboratories may not be directly comparable.^6,20^

This cross-protocol analysis describes the distribution of SARS-CoV-2 VL at the time of COVID-19 diagnosis for over 1600 placebo recipients from 4 phase 3 COVID-19 vaccine trials conducted in partnership with the COVID-19 Prevention Network (CoVPN).^21,22^ The data span 8 countries, and waves of infection attributable to the ancestral variant and 9 others. The harmonized COVID-19 definitions and timing of specimen collection across this diverse cohort allowed us to systematically examine factors of variability in SARS-CoV-2 VL at diagnosis prior to any immunization. It also allowed for an assessment of the ability of VL to predict COVID-19 severity in a largely outpatient disease context.

## Methods

### Participants

This cross-protocol analysis included participant-level data from the placebo groups of 4 randomized, placebo-controlled, phase 3 COVID-19 vaccine efficacy trials.^22,23,24,25,26^ The trials (herein referenced by study sponsor: [1] Moderna, [2] Janssen, [3] AstraZeneca, and [4] Novavax) were conducted under a US government–funded program, with the CoVPN providing organizational leadership and infrastructure.^21^ The trials featured harmonized protocols, including primary end points, with start dates from July 27 to December 27, 2020, and primary analysis data cutoffs from March 26 to July 30, 2021. Trial sites were located in the US, Brazil, South Africa, Colombia, Argentina, Peru, Chile, and Mexico,^22^ comprising a diverse study population and varying epidemiological trends.^27^ Of note, the Moderna, AstraZeneca, and Novavax trials were conducted primarily in the US (exclusively US for Moderna), while the Janssen trial spanned 8 countries across 3 continents. Local or central institutional review board and/or ethics committee approvals were obtained by each site participating in the 4 trials.^23,24,25,26^

The study cohort consisted of participants from the placebo groups of the trials who were SARS-CoV-2 negative at enrollment (based on polymerase chain reaction (PCR) and antinucleocapsid serology, or antinucleocapsid serology alone for the AstraZeneca trial) and who were diagnosed with COVID-19 meeting the primary end point definitions (eMethods in Supplement 1) during the blinded phase of the trial. This cohort reflects the COVID-19 experience for the immunologically naive early in the pandemic.

Primary end point COVID-19 was defined as independently adjudicated COVID-19 occurring at least 14 days (≥7 days for Novavax) following the last placebo injection, as detailed in the original trial publications^23,24,25,26^ and the eMethods in Supplement 1. Broadly, criteria included a positive molecular test (eg, PCR) accompanied by systemic and/or respiratory symptoms; severe COVID-19 was characterized by more substantial symptoms (eMethods in Supplement 1).

The analysis cohort consisted of study cohort participants with VL measurements at COVID-19 diagnosis, including participants who were PCR-negative (0 VL) on their protocol-defined COVID-19 diagnosis date (described in next section). For the Moderna trial, the analysis cohort was further limited to participants who tested negative through day 57 by reverse transcriptase PCR (RT-PCR) and antinucleocapsid serology assay, and PCR positive on the protocol-defined date of COVID-19 diagnosis.

### Viral Load Measurements

The primary outcome for this analysis was SARS-CoV-2 VL at COVID-19 diagnosis, defined by VL measured from the nasal and/or nasopharyngeal (NP) swab closest to protocol-defined COVID-19 onset. COVID-19 onset was defined as the date of first positive SARS-CoV-2 PCR test (AstraZeneca), symptom onset (Janssen), the earlier of the 2 (Novavax), or the later of the 2 (Moderna) (eMethods in Supplement 1). Therefore, for some participants it was possible for a PCR test on the date of COVID-19 diagnosis to be negative. Trial was included as a covariate in all analyses, as RT-PCR and VL quantification were performed at different laboratories and using different assays across the trials (see eMethods in Supplement 1).

Symptom-driven PCR testing varied by protocol: Moderna and AstraZeneca brought participants into clinics for confirmatory PCR testing of nasal and/or NP swabs within 1 to 3 days of symptom onset; Janssen and Novavax provided nasal swabs for home collection at symptom onset. Days since COVID-19 onset was included in all analyses to account for timing differences in sample collection. Protocol-specific central laboratories derived VL using validated RT-PCR assays with concurrently run standards for conversion to log_10_ copies/mL (eMethods in Supplement 1).

### Variant Identification

Sequencing was attempted for all infections by protocol-specific laboratories and successful sequences were lineage-typed to identify the viral variant. Full genomes from Janssen and Novavax infections were lineage-typed with the PANGOLIN tool,^28^ whereas spike-only sequences from Moderna and AstraZeneca were assigned a World Health Organization (WHO) variant label using a tool we developed for this purpose (eMethods in Supplement 1). Specimens with sequences that met one of the WHO-named variant definitions were classified as such; the remaining sequences were from the A.1 and B.1 lineages and classified as ancestral.^29^ Specimens without sequencing data were considered to have missing variant.

As an alternative to variant classification, for samples with sequencing data, spike Hamming distances were calculated as the number of amino acid positions differing from the Wuhan-Hu-1 ancestral strain (GenBank accession number NC_045512).^30^

### Statistical Analyses

Linear regression was used to assess the association of baseline participant characteristics, exposure risk factors, and disease characteristics with log_10_ VL at COVID-19 diagnosis. Covariate definitions are provided in the eMethods in Supplement 1. A multivariate model included prespecified covariates based on literature review: age at baseline, sex assigned at birth, self-identified race, ethnicity, baseline self-reported comorbidities associated with high risk of severe COVID-19, country of residence, variant, COVID-19 severity, days since protocol-defined COVID-19 onset, and trial.

Multiple imputation was used to ascribe missing variants using the population proportion of diagnosed infections attributed to each variant within 2 weeks of the date of COVID-19 onset, based on country- or state-specific genomic surveillance data from the Global Initiative on Sharing All Influenza Data (GISAID).^31^ Results were combined across 20 imputed data sets using Rubin rules (eMethods in Supplement 1).^32^ The Holm method^33^ was used to control the familywise error rate at 0.05 across univariate analyses and separately among the variables in the multivariate model.

Univariate, sensitivity, and exploratory analyses were also performed to explore the robustness of our conclusions (eMethods in Supplement 1). The multivariate model was fit without imputation to the subset of participants with viral sequence data, to participants infected with the ancestral variant, and using the spike Hamming distance of the sequence to the ancestral strain in lieu of variant. Additionally, a generalized additive model (GAM) extension of the multivariate model was fit. The GAM model included country-specific smoothed calendar time trends to account for local epidemic dynamics.^34,35^ Finally, the multivariate analysis was repeated for the subsets of participants who had nonzero VL at diagnosis, who enrolled in the US, and who enrolled in the Janssen trial. By examining more homogenous populations, we aimed to circumvent the effects of confounding variables.

For the Janssen trial, which captured the largest number of COVID-19 events and had the most intensive post–COVID-19 diagnosis specimen collection, log_10_ VL at diagnosis and area under the 28-day log_10_ VL curve (corresponding to the mean VL) were evaluated for their ability to predict severe COVID-19, with and without the full set of baseline participant characteristics and covariates. Risk of severe COVID-19 was estimated using super learning, and prediction performance was measured using the cross-validated area under the receiver operating characteristic (ROC) curve (AUC) (eMethods in Supplement 1).^36,37,38^ Two-sided *P* < .05 was deemed statistically significant. Statistical analysis was performed using R version 4.0.4 (R Project for Statistical Computing) from November 2022 to June 2023.

## Results

### Participant Demographics and Disease Characteristics

**Figure 1.**
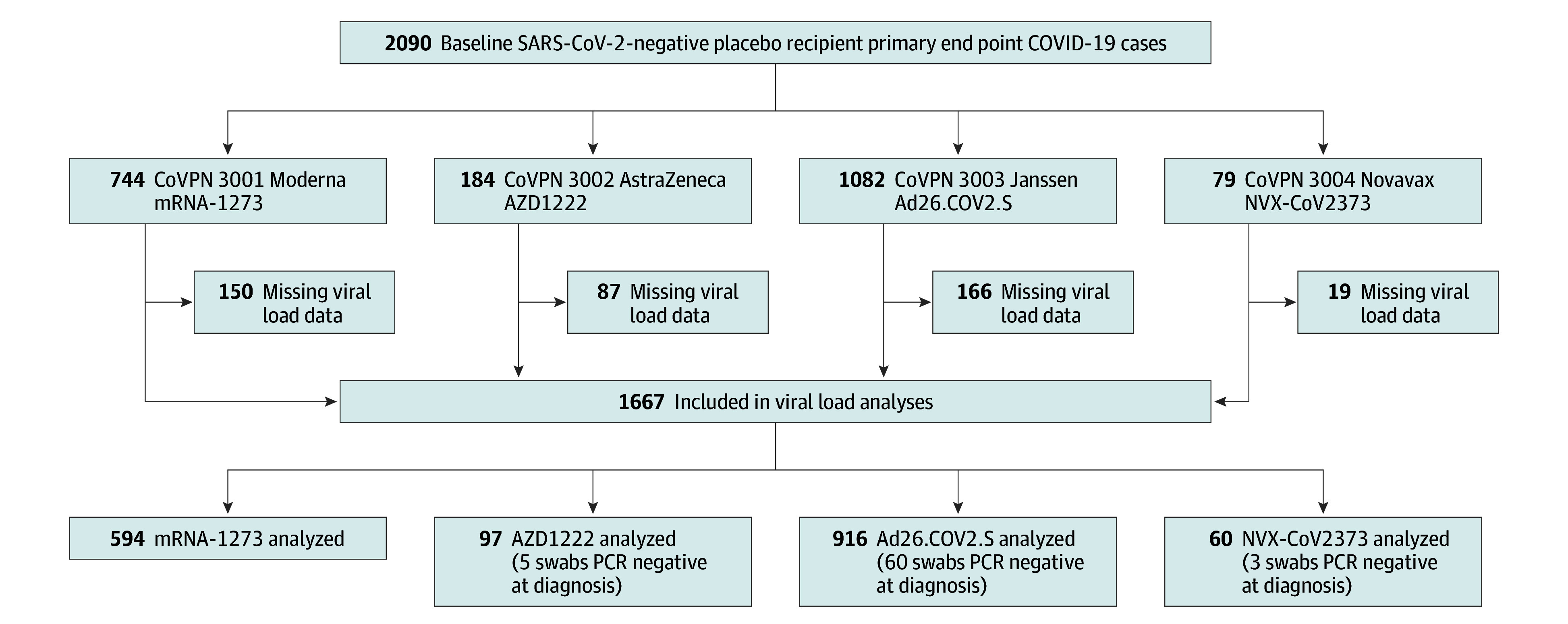
Overview of Study and Analysis Cohorts The study cohort consisted of participants randomized to the placebo group of each of the 4 US government–sponsored, phase 3 vaccine efficacy trials who were SARS-CoV-2 negative at baseline and went on to have a primary end point COVID-19 infection. Those participants with viral load data from diagnosis (ie, the first illness-associated polymerase chain reaction [PCR] test) were included in the analysis cohort, including those with negative PCR swabs. CoVPN indicates COVID Vaccine Prevention Network.

The analysis cohort included 1667 participants, with Moderna (n = 594 [35.6%]) and Janssen (n = 916 [54.9%]) contributing the majority (Figure 1). In total, 886 participants (53.1%) were male; 995 (59.7%) were enrolled in the US; the mean (SD) age was 46.7 (14.7) years, 204 (12.2%) were 65 years or older; 196 (11.8%) were American Indian or Alaska Native, 41 (2.5%) were Asian, 150 (9.0%) were Black or African American, 110 (6.6%) reported multiple races, 1112 (66.7%) were White, 13 (2.2%) were other race; and 762 (45.7%) were Hispanic or Latino. Differences in the number of cases between trials were affected by the sizes of the placebo groups and epidemiological trends during follow-up.^27^

Baseline and disease characteristics are summarized by trial in the Table and eTable 1 in Supplement 1; 577 participants (34.6%) had preexisting comorbidities, and 1226 (73.5%) were categorized as overweight or obese at baseline. Among these participants with symptomatic COVID-19, for 263 (15.8%) the disease was classified as severe, although hospitalization rates were generally low (less than 0.2% in the placebo group of each contributing trial).^23,24,25,26,39^ VL was typically measured within 1 day of COVID-19 onset; sequences and infecting variants were available for 1323 participants (79.4%); of those, 857 (65.5%) corresponded to the ancestral variant. Nine other variants (Alpha, Beta, Gamma, Epsilon, Zeta, Iota, Delta, Lambda, and Mu) were detected at frequencies less than 10%. The Janssen trial included the greatest number of countries and variants. Because of this and other epidemiological and trial factors, country, variant, and trial were confounded in the data set.

**Table.** Baseline Characteristics and Clinical Characteristics of Placebo Recipients Who Developed COVID-19 in 1 of 4 CoVPN Phase 3 COVID-19 Vaccine Efficacy Trials, With SARS-CoV-2 Viral Load Measured at COVID-19 Diagnosis

Characteristic	Parent protocol				
	Moderna (n = 594)	AstraZeneca (n = 97)	Janssen (n = 916)	Novavax (n = 60)	Total (n = 1667)
Sex assigned at birth, No. (%)					
Female	293 (49.3)	33 (34.0)	417 (45.5)	38 (63.3)	781 (46.9)
Male	301 (50.7)	64 (66.0)	499 (54.5)	22 (36.7)	886 (53.1)
Country, No. (%)					
Argentina	0	0	95 (10.4)	0	95 (5.7)
Brazil	0	0	169 (18.4)	0	169 (10.1)
Chile	0	7 (7.2)	9 (1.0)	0	16 (1.0)
Colombia	0	0	192 (21.0)	0	192 (11.5)
Mexico	0	0	8 (0.9)	4 (6.7)	12 (0.7)
Peru	0	20 (20.6)	84 (9.2)	0	104 (6.2)
South Africa	0	0	84 (9.2)	0	84 (5.0)
US	594 (100.0)	70 (72.2)	275 (30.0)	56 (93.3)	995 (59.7)
Self-reported race, No. (%)^a^					
American Indian or Alaska Native^b^	4 (0.7)	16 (16.5)	172 (18.8)	4 (6.7)	196 (11.8)
Asian	23 (3.9)	0	14 (1.5)	4 (6.7)	41 (2.5)
Black or African American	29 (4.9)	6 (6.2)	110 (12.0)	5 (8.3)	150 (9.0)
Multiple	7 (1.2)	4 (4.1)	99 (10.8)	0	110 (6.6)
Not reported	10 (1.7)	1 (1.0)	33 (3.6)	1 (1.7)	42 (2.7)
White	508 (85.5)	70 (72.2)	488 (53.3)	46 (76.7)	1112 (66.7)
Other	13 (2.2)	0	0	0	13 (0.8)
Ethnicity, No. (%)					
Hispanic or Latino	134 (22.6)	36 (37.1)	580 (63.3)	12 (20.0)	762 (45.7)
Not Hispanic or Latino	458 (77.1)	60 (61.9)	319 (34.8)	48 (80.0)	885 (53.1)
Not reported	2 (0.3)	1 (1.0)	17 (1.9)	0	20 (1.2)
Age category, No. (%), y					
18-29	70 (11.8)	18 (18.6)	168 (18.3)	18 (30.0)	274 (16.4)
30-39	108 (18.2)	14 (14.4)	122 (13.3)	11 (18.3)	255 (15.3)
40-49	141 (23.7)	25 (25.8)	226 (24.7)	10 (16.7)	402 (24.1)
50-64	194 (32.7)	28 (28.9)	292 (31.9)	18 (30.0)	532 (31.9)
≥65	81 (13.6)	12 (12.4)	108 (11.8)	3 (5.0)	204 (12.2)
BMI category, No. (%)					
<18.5	3 (0.5)	1 (1.0)	6 (0.7)	0	10 (0.6)
18.5-<25.0	115 (19.4)	24 (24.7)	263 (28.7)	23 (38.3)	425 (25.5)
≥25.0	472 (79.5)	71 (73.2)	646 (70.5)	37 (61.7)	1226 (73.5)
Missing	4 (0.7)	1 (1.0)	1 (0.1)	0	6 (0.4)
COVID-19 comorbidities, No. (%)	138 (23.2)	61 (62.9)	346 (37.8)	32 (53.3)	577 (34.6)
Severe COVID-19 symptoms, No. (%)	81 (13.6)	4 (4.1)	175 (19.1)	3 (5.0)	263 (15.8)
Days since COVID-19 onset, No. (%)^c^					
−1	25 (4.2)	0	0	0	25 (1.5)
−2	10 (1.7)	0	0	0	10 (0.6)
0	554 (93.3)	97 (100.0)	263 (28.7)	12 (20.0)	926 (55.5)
1	3 (0.5)	0	312 (34.1)	12 (20.0)	327 (19.6)
2	2 (0.3)	0	149 (16.3)	22 (36.7)	173 (10.4)
3	0	0	96 (10.5)	12 (20.0)	108 (6.5)
4	0	0	96 (10.5)	2 (3.3)	98 (5.9)
Infecting variant: No. (%)					
Ancestral	435 (73.2)	43 (44.3)	376 (41.0)	13 (21.7)	867 (52.0)
Alpha	0	9 (9.3)	25 (2.7)	24 (40.0)	58 (3.5)
Beta	0	0	49 (5.3)	1 (1.7)	50 (3.0)
Gamma	1 (0.2)	1 (1.0)	105 (11.5)	3 (5.0)	110 (6.6)
Epsilon	13 (2.2)	5 (5.2)	16 (1.7)	3 (5.0)	37 (2.2)
Zeta	1 (0.2)	0	74 (8.1)	1 (1.7)	76 (4.6)
Iota	0	0	4 (0.4)	2 (3.3)	6 (0.4)
Delta	0	0	7 (0.8)	0	7 (0.4)
Lambda	0	16 (16.5)	43 (4.7)	0	59 (3.5)
Mu	0	0	53 (5.8)	0	53 (3.2)
No sequence	144 (24.2)	23 (23.7)	164 (17.9)	13 (21.7)	344 (20.6)

Abbreviations: BMI, body mass index (calculated as weight in kilograms divided by height in meters squared); CoVPN, COVID Vaccine Prevention Network.

^a^ Self-reported race is defined across all clinical sites. Participants were asked to select all applicable categories, including “Other”; Multiple indicates more than one self-reported category; Not reported indicates a missing response.

^b^ Indigenous people from South America were classified together with the American Indian or Alaska Native US and Mexico demographic according to the FDA definition (American Indian or Alaska Native: A person having origins in any of the original peoples of North and South America (including Central America), and who maintains tribal affiliation or community attachment). In this analysis, the Moderna, AstraZeneca, Janssen, and Novavax trials included 4, 1, 5, and 4 participants, respectively, who identified as American Indian or Alaskan Native from North America.

^c^ Days since COVID-19 onset is defined as the number of calendar days between protocol-defined onset of COVID-19 and the specimen collection corresponding to diagnosis. Negative days since onset in Moderna implies the positive swab was obtained before qualifying symptom onset.

### Variability in Viral Load

**Figure 2.**
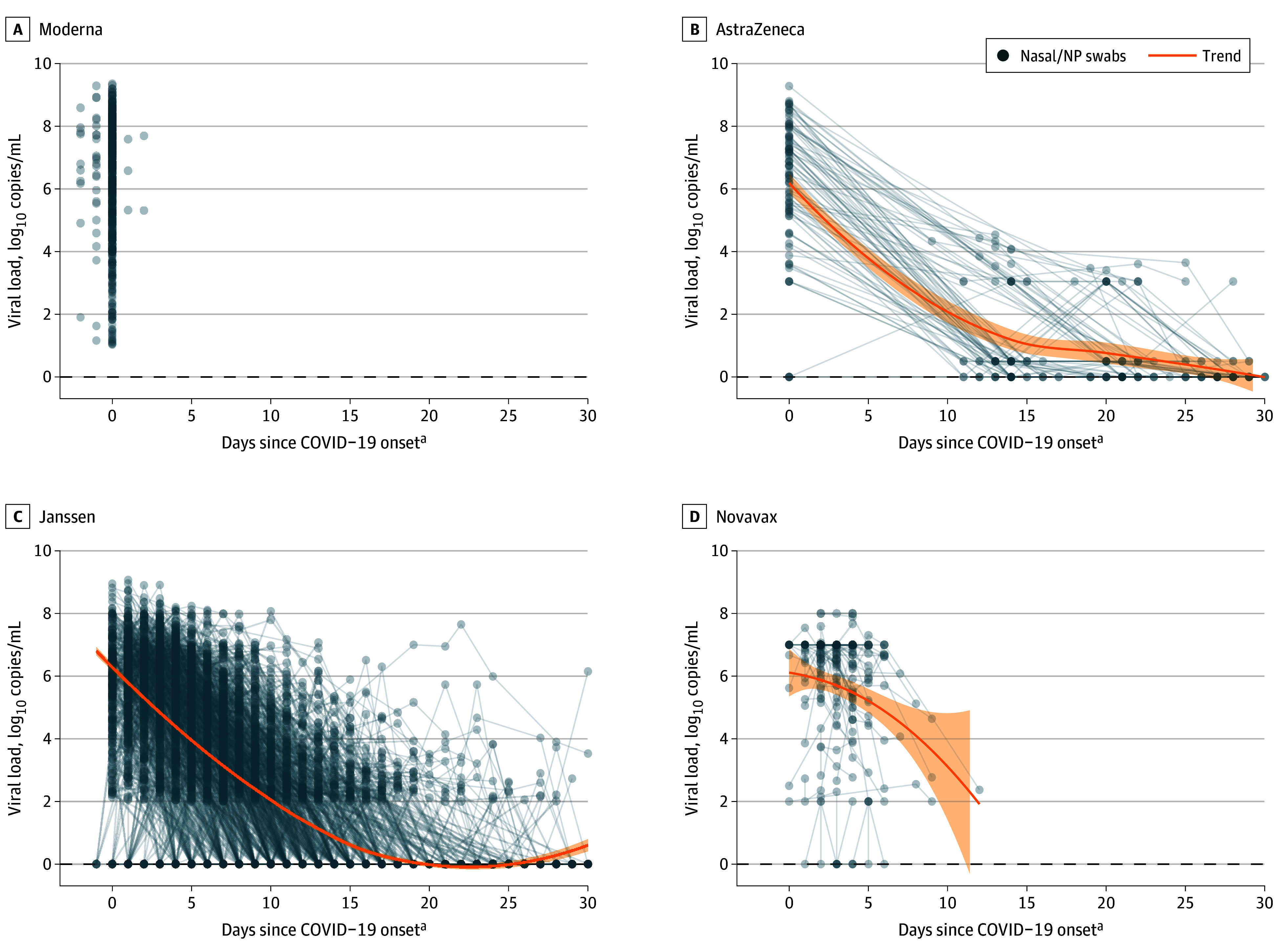
Protocol-Specific Individual-Level SARS-CoV-2 Viral Load Data Over Illness Visits Blue dots denote individual viral load values based on nasal and/or nasopharyngeal (NP) swabs; gray lines connect results from the same participant. Orange curves are smooth estimates using locally estimated scatterplot smoothing and summarize viral load trends based on nasal and/or NP swabs. Moderna collected saliva swabs post–COVID-19 onset, which are not shown given the focus on viral load based on nasal/NP swabs. ^a^COVID-19 onset was defined in each parent protocol: the date of first positive polymerase chain reaction (PCR) test (AstraZeneca), symptom onset (Janssen), the earlier of the 2 (Novavax), or the later of the 2 (Moderna). Thus, for Janssen and Moderna some PCR-positive tests prior to COVID-19 onset were observed.

For most participants, VL was highest near COVID-19 onset and declined over time, with considerable interindividual variation (Figure 2). All protocols measured VL with nasal and/or NP swabs at diagnosis, but collection method and frequency differed thereafter (eMethods in Supplement 1). Accordingly, the present analysis focuses on VL at diagnosis.

**Figure 3.**
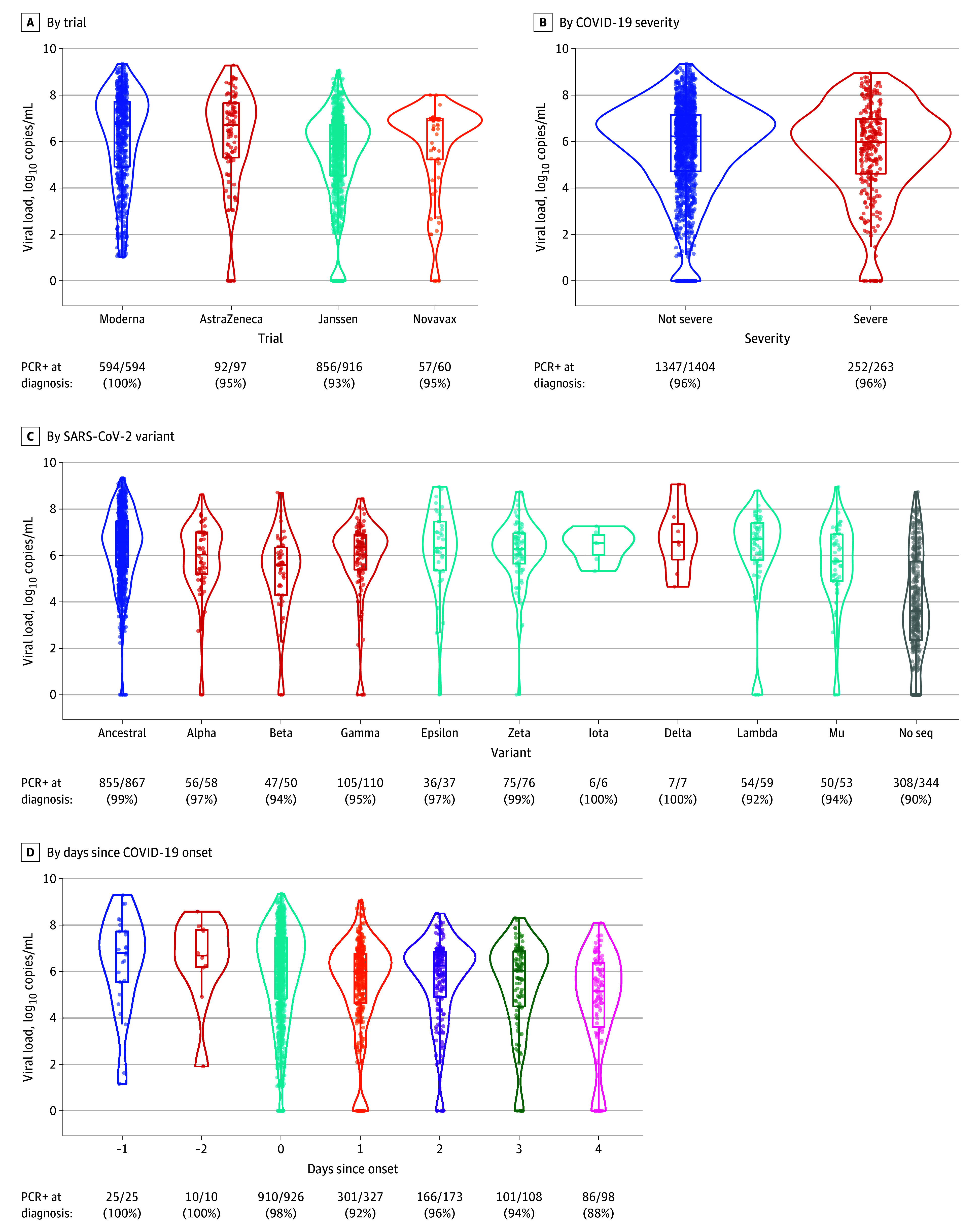
SARS-CoV-2 Viral Load in Nasopharyngeal Swab at COVID-19 Diagnosis by Trial, COVID-19 Severity, SARS-CoV-2 Variant, and Days Since COVID-19 Onset At the bottom of each panel, the number and percentage of participants with detectable viral load (>0 copies/mL) at diagnosis are provided. In panel C, colors indicate the highest level of World Health Organization designation: dark blue for the ancestral variant, red for variants of concern, light blue for variants of interest, and gray for those missing sequence. In panel D, COVID-19 onset was defined in each parent protocol: the date of first positive polymerase chain reaction (PCR) test (AstraZeneca), symptom onset (Janssen), the earlier of the 2 (Novavax), or the later of the 2 (Moderna). Thus, for Janssen and Moderna there were some PCR-positive tests prior to COVID-19 onset.

VL at diagnosis was highly variable, with a median (IQR) of 6.18 (4.66-7.12) log_10_ copies/mL. From the 3 protocols that provided undetectable (0) VL results, 68 of 1073 participants (6.3%) in the analysis cohort were in this category. Importantly, these participants at some point did meet the primary end point definition of symptomatic PCR-confirmed COVID-19, and therefore had another (positive) PCR swab associated with this infection. Distributions of log_10_ VL by trial, COVID-19 severity, SARS-CoV-2 variant, and days since disease onset are summarized in Figure 3, and univariate associations are summarized in eTable 2 in Supplement 1. Although viral sequence data would naturally be missing for participants with 0 or very low VL (given that amplification of viral RNA is necessary for sequencing), the 344 (20.6%) missing sequences included a wide range of VLs (Figure 3C).

### Multivariate Model Associations With Viral Load

**Figure 4.**
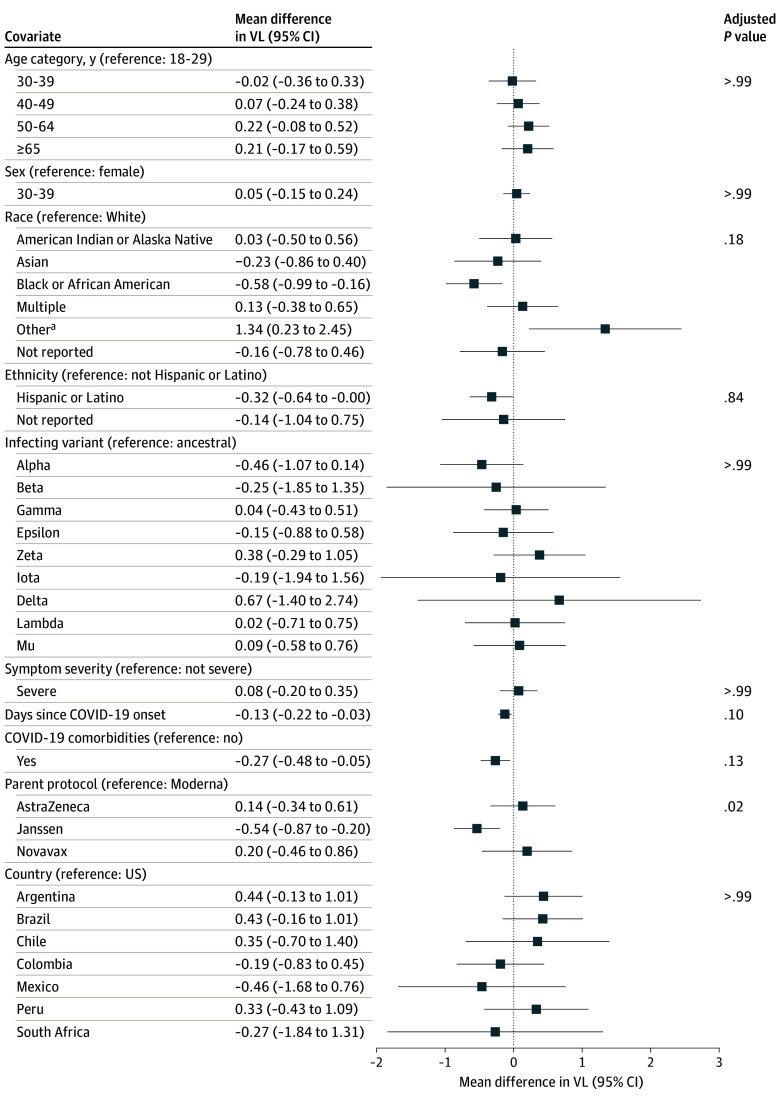
Estimated Mean Differences in SARS-CoV-2 Viral Load in Nasal and/or Nasopharyngeal (NP) Swabs at COVID-19 Diagnosis, Based on Multivariate Model Forest plot illustrating estimated mean difference in log_10_ copies/mL SARS-CoV-2 viral load between groups defined by participant or COVID-19 characteristics; 95% CIs and Holm-adjusted *P* values are provided.

The multivariate model identified few independent factors associated with VL at diagnosis (Figure 4). Trial showed the strongest association (*P* = .02): participants in the Janssen trial had 0.54 log_10_ copies/mL lower mean VL compared to those in the Moderna trial (95% CI: 0.20 to 0.87 log_10_ copies/mL lower).

Although race did not show a statistically significant association overall, mean VL was 0.58 (95% CI, 0.99-0.16) log_10_ copies/mL lower among those who reported as Black or African American vs White, and 1.34 log_10_ (95% CI, 95% CI, 0.23-2.45) copies/mL higher for the 13 Moderna participants who reported other race and ethnicity vs White.

COVID-19 severity was not significantly associated with VL. There were likewise no differences in VL between countries or infecting variants. COVID-19 severity was not significantly associated with VL. Considering SARS-CoV-2 variants, the highest VL was observed for the 7 Delta infections, although the 95% CI for the mean difference was wide (0.67 log_10_ copies/mL higher vs ancestral virus; 95% CI, 1.40 lower to 2.74 higher log_10_ copies/mL). In total, the multivariate model explained just 5.9% of the variability in VL, indicating considerable unexplained variability even after accounting for all variables in the model.

### Hamming Distance and Sensitivity Analyses

The multivariate model was also fit to participants with available sequence data using spike Hamming distances instead of variant; the analysis restricted attention to participants with sequence data. There was no association found between VL and Hamming distance, with a nonsignificant 0.01 log_10_ copies/mL lower mean VL per additional nucleotide difference from the ancestral strain (95% CI, 0.04 lower to 0.01 higher log_10_ copies/mL) (eMethods, eFigure 1, eTable 4 in Supplement 1).

The complete case analysis, wherein the multivariate model was fit to the subset of 1323 participants with sequence data (ie, without imputation), yielded similar results to the primary analysis (eFigure 2 in Supplement 1). Participants in the Janssen trial had 1.06 log_10_ copies/mL lower mean VL vs participants in the Moderna trial (95% CI, 1.35-0.76 log_10_ copies/mL lower; *P* < .001).

In an analysis restricted to participants in the analysis cohort with PCR-positive results at diagnosis, race was the only significant association (*P* = .006): compared with White-identifying participants, Black or African American participants had 0.59 log_10_ copies/mL lower mean VL (95% CI, 0.94 to 0.23 lower log_10_ copies/mL), and other race was associated with a 1.32 log_10_ copies/mL higher mean VL (95% CI, 0.39 to 2.29 higher log_10_ copies/mL) (eFigure 3 in Supplement 1). In this analysis, trial did not show a significant association with VL.

For the sensitivity analyses that separately accounted for local temporal trends, restricted to US participants, or restricted to ancestral SARS-CoV-2 infections, qualitative conclusions were unchanged. In each analysis, participants in the Janssen trial exhibited lower mean VL at diagnosis, but no associations were statistically significant (eFigures 4-6 in Supplement 1). Moreover, the sensitivity analysis restricted to Janssen participants did not identify any factors significantly associated with VL (eFigure 7 in Supplement 1).

### Viral Load as a Predictor of Severe COVID-19

Among 916 participants in the analysis cohort from the Janssen trial, neither VL at diagnosis nor area under the VL curve (AUC-VL), interpreted as the mean VL over days 1 to 28 post–COVID-19 diagnosis, predicted COVID-19 severity (cv-AUC, 0.52 [95% CI 0.47 to 0.57]; and AUC-VL cv-AUC, 0.49 [95% CI, 0.42 to 0.57]) (eMethods, eFigure 8 in Supplement 1). Incorporating baseline participant characteristics, characteristics of COVID-19 diagnosis, and VL measurements were associated with improved predictive performance (cv-AUC, 0.71 [95% CI, 0.67 to 0.75]); however, variable importance measures suggest the dominant predictors were race and variant, and neither VL predictor was among the top 10 in the model (eFigure 9 in Supplement 1). Moreover, VL at diagnosis was not associated with improved predictions when added to a model including other baseline characteristics (eFigure 11 in Supplement 1).

## Discussion

In a large and diverse cohort of immunologically naive participants with acute COVID-19, we observed considerable variability in VL at diagnosis, only a small fraction of which was explained by participant characteristics. Although not a statistically significant result, we estimated the highest mean VL among participants infected with Delta, consistent with previous literature.^40,41^ The strongest measured association with VL in our study was that with trial, which suggests timing of specimen collection or other factors associated with the specimen collection, storage, or VL assays across protocols may have influenced VL measurements.^42,43,44,45^ These results should temper expectations of future research comparing VL across trials or settings, especially given increasing diversity in preexisting immunity.

Intriguingly, we did not find an association between VL at diagnosis and severe COVID-19 in this largely outpatient setting. Neither VL at diagnosis nor averaged over days 1 to 28 post COVID-19 onset predicted severe disease. This may reflect that severe COVID-19 is typically caused by lower respiratory tract infection, which may not be detected by nasal and/or nasopharyngeal swabbing, and in the case of VL at diagnosis, may also reflect that severe disease may take days or weeks to fully manifest. This result contrasts with previous studies which have documented associations between VL at diagnosis^46,47,48,49,50^ or during the second week of infection^40^ and severe disease. Importantly, however, prior studies were conducted primarily in hospitalized populations, and our study is unique in its capture of individuals with COVID-19 symptoms in a primarily outpatient context. The observed variability in VL among these participants may undermine the use of VL as a proxy for clinical outcomes in this population. The utility of VL end points in future trials of prophylactic and therapeutic interventions should also be considered carefully given this result.

There are several possible explanations for the lower mean VL observed in the Janssen trial. Swabs from this trial were self-collected, which may have resulted in poorer sample quality. While Novavax also used self-collection, there were fewer samples from this trial, potentially obscuring statistical significance. It is also noteworthy that Janssen samples underwent RT-PCR diagnostic testing at study sites or at a local central laboratory and were then frozen and shipped to the central virology laboratory (University of Washington) to undergo confirmatory testing and VL quantification, resulting in at least 2 freeze-thaw cycles. It has been shown that multiple freeze-thaw cycles can degrade RNA specimens, potentially more substantially for low VL specimens, which would further decrease low VL measurements.^51^ Also, while our analysis excluded participants PCR-positive or seropositive at baseline, participants who were previously infected but seroreverted prior to enrollment may have been included; these individuals would likely have lower VL and be overrepresented in the Janssen trial (conducted later in the pandemic). Importantly, however, whether there is clinical importance to the estimated 0.54 log_10_ copies/mL lower mean VL in the Janssen trial is also uncertain.

### Limitations

Our study has limitations. Differences across protocols in collection schedules and methods, specimen types, and timing after the onset of symptoms limited our analyses to VL measurements from a single time point. Furthermore, nasal swabs are subject to heterogeneity; less variability may have been observed if blood samples were analyzed. We were unable to evaluate any association between VL and transmission, because secondary cases were not assessed, and differences in available VL data across protocols limited our analyses to primary end point infections among immunologically naive participants, although analyses assessing the association between COVID-19 vaccination with VL will be reported separately. VL and COVID-19 severity analyses were limited to a single trial (Janssen), although it did include the largest number of COVID-19 events, countries, variants, and severe disease events among the trials included. Furthermore, our analyses were limited to the harmonized, adjudicated secondary end point definition of severe COVID-19; this included patients exhibiting prespecified signs and symptoms, most of whom were not hospitalized. It is also worth noting that our study does not characterize viral load in asymptomatic individuals; however, contact-tracing and household studies suggest that asymptomatic cases may transmit at a lower rate.^52,53,54,55,56,57^ Additionally, even with prompt PCR testing shortly after symptom onset, the collected specimens likely missed the peak VL and reflect the declining phase of the VL trajectory.^58^

Statistical comparisons were also limited by the data available. Importantly, laboratories and assays measuring VL differed across protocols, and were thus confounded with trial, preventing an analysis that adjusts for or stratifies by assay type. Because most participants in the analysis cohort who resided outside the US were enrolled in the Janssen trial, primary analyses could not fully disentangle country and study associations with VL. However, the fact that sensitivity analyses restricted to the Janssen trial detected no VL differences between countries, while US-restricted analyses still detected differences in VL between protocols, suggests that the variable with the strongest association with VL was indeed trial.

## Conclusions

The large variability in VL that we observed in this secondary cross-protocol analysis has important implications. Studies evaluating mucosal COVID-19 vaccines, which are thought to potentially affect transmission as measured by VL, are especially relevant. Future studies will likely be conducted among even more diverse settings, including participants with a wide variety of infection and vaccination histories. Studies including participants with both symptomatic and asymptomatic SARS-CoV-2 infection are expected to have even greater variability in VL than what was observed here among exclusively symptomatic participants. Standardization in prompts for testing, collection, processing, storage, and assaying of specimens will be critical to minimize variability and allow the effects of interventions and other exposures to be evaluated.

## Notes

Supplement 1.
eMethods.

Supplement 2.
Nonauthor Collaborators

Supplement 3.
Data Sharing Statement
