# Supplementary material for: SARS-CoV-2 Viral Load in the Nasopharynx at Time of First Infection Among Unvaccinated Individuals: A Secondary Cross-Protocol Analysis of 4 Randomized Trials
Source: JAMA Netw Open. 2024 May 23;7(5):e2412835. doi: 10.1001/jamanetworkopen.2024.12835 (PMC11117088; doi:10.1001/jamanetworkopen.2024.12835)
Supplement: Supplement 1. — eMethods. [file jamanetwopen-e2412835-s001.pdf]

# Supplemental Online Content

Fisher LH, Kee JJ, Liu A, et al; for the COVID-19 Prevention Network (CoVPN). SARS-CoV-2 Viral Load in the Nasopharynx at Time of First Infection Among Unvaccinated Individuals. *JAMA Netw Open.* 2024;7(5):e2412835. doi:10.1001/jamanetworkopen.2024.12835


**eMethods**

**eTable 1.** Additional Baseline Characteristics

**eTable 2.** Univariate Linear Regression Results for Placebo Infections

**eTable3.** Comparison of Model Results From Three Univariate Analysis of Infecting Variant

**eFigure 1.** Scatter Plot of Viral Load (log10 copies/mL) by Hamming Distance of Spike Sequence to Ancestral SARS-CoV-2, for Those Placebo Infections With Successful Sequencing

**eTable 4.** Multivariate Linear Regression of log10 Viral Load at Diagnosis Using Hamming Distance

**eFigure 2.** Estimated Mean Differences in SARS-CoV-2 Viral Load in Nasal/NP Swab at COVID-19 Diagnosis Among Those With Successful Variant Calls (N = 1,323; Adjusted R2 = 0.106)

**eFigure 3.** Estimated Mean Differences in SARS-CoV-2 Viral Load in Swabs at COVID-19 Diagnosis on the Subset of Participants With Quantifiable Viral Load at Diagnosis, Imputing Missing Variants

**eFigure 4.** (A) Summary of Parametric Results From GAM Analysis With Country-Specific Temporal Regression Splines (N = 1,667; Adjusted R2 = 0.081) and (B) Estimated Country-Level Temporal Smoothers

**eFigure 5.** Multivariate Linear Regression of log10 Viral Load at Diagnosis on the Subset of Participants Infected With the Ancestral Variant Only (N = 867; Adjusted R2 = 0.104)

**eFigure 6.** Estimated Mean Differences in SARS-CoV-2 Viral Load in Swabs at COVID-19 Diagnosis on the Subset of Participants Living in the US, Imputing Missing Variants (N=995; Adjusted R2 = 0.047)

**eFigure 7.** Estimated Mean Differences in SARS-CoV-2 Viral Load in Swabs at COVID-19 Diagnosis on the Subset of Placebo Participants Enrolled in the Janssen trial, Imputing Missing Variants (N=916)

**eFigure 8.** Summary of the Utility of Log Viral Load at Diagnosis (A) and the Area Under the VL Trajectory Curve (B) in Predicting Severe COVID-19

**eFigure 9.** Marginal Variable Importance Measures for All Available Baseline Characteristics and Covariates Collected Around the Time of Infection

**eFigure 10.** Summary of Multivariate Predictors of COVID-19 Severe Disease

**eFigure 11.** Estimated Conditional Variable Importance Measures and 95% CI for the Features in the Prediction of Severe COVID-19

**eReferences**


This supplemental material has been provided by the authors to give readers additional information about their work.

© 2024 Fisher LH et al. *JAMA Network Open.*

# eMethods

## CoVPN Study and Covariate Harmonization

The Moderna, AstraZeneca, Janssen, and Novavax trials were designed to be harmonized, but minor differences were present. Important aspects of the trials are summarized in **eMethods Table 1**.

**eTable 1. Summary of protocol-specific definitions, including date of COVID-19 onset and COVID-19 symptoms that triggered illness visit testing.**

| | Moderna | AstraZeneca | Janssen | Novavax |
|---|---|---|---|---|
| **COVID-19 Case definition** | • The participant must have experienced at least TWO of the following systemic symptoms: Fever (≥38ºC), chills, myalgia, headache, sore throat, new olfactory and taste disorder(s); OR<br>• The participant must have experienced at least ONE of the following respiratory signs/symptoms: cough, shortness of breath or difficulty breathing, OR clinical or radiographical evidence of pneumonia; AND<br>• The participant must have at least one NP swab, nasal swab, or saliva sample (or respiratory sample, if hospitalized) positive for SARS-CoV-2 by RT-PCR | Participant must have RT-PCR-confirmed SARS-CoV-2 and meet the following criteria at any point from their initial illness visit at the site (Day 1) through their second illness visit (Day 14):<br>1 One or more Category A findings<br>OR<br>2 Two or more Category B findings<br><br>Category A:<br>• Pneumonia diagnosed by chest x-ray, or computed tomography scan<br>• Oxygen saturation of ≤94% on room air or requiring either new initiation or escalation in supplemental O2<br>• New or worsening dyspnea/shortness of breath<br><br>Category B:<br>• Fever >100°F (>37.8°C) or feverishness<br>• New or worsening cough<br>• Myalgia/muscle pain<br>• Fatigue that interferes with activities of daily living<br>• Vomiting and/or diarrhea (only one finding to be counted toward endpoint definition)<br>• Anosmia and/or ageusia (only one finding to be counted toward endpoint definition) | PCR or NAAT confirmation of SARS-CoV-2 infection AND ≥2 of the following symptoms (new or worsening): fever or chills, cough, heart rate ≥90 beats/minute, muscle or body pain, headache, new loss of taste or smell, sore throat, red or bruised-looking feet or toes, nausea, vomiting, or diarrhea; or one or more of the following signs or symptoms: shortness of breath, respiratory rate >20 breaths/minute, clinical or radiologic evidence of pneumonia, deep vein thrombosis, or abnormal oxygen saturation (but above 93%). | PCR-confirmed COVID-19, either mild (≥ 1 of the following: subjective or objective fever or new onset cough; or ≥ 2 of the following: new onset or worsening of shortness of breath or difficulty breathing compared to baseline, new onset fatigue, new onset generalized muscle or body aches, new onset headache, new loss of taste or smell, acute onset of sore throat, congestion or runny nose, new onset nausea, vomiting or diarrhea) OR moderate (≥1 of the following: fever ≥38.4°C for ≥3 days, any evidence of significant lower respiratory tract infection [shortness of breath or breathlessness or difficulty breathing with or without exertion greater than baseline], tachypnea [24 to 29 breaths per minute at rest], SpO2 94% to 95% on room air, abnormal chest X-ray or chest computerized tomography consistent with pneumonia or lower respiratory tract infection, adventitious sounds on lung auscultation [e.g., crackles/rales, wheeze, rhonchi, pleural rub, stridor]. |

© 2024 Fisher LH et al. *JAMA Network Open.*

| | | | | |
|---|---|---|---|---|
| **Severe COVID-19 case definition** | Meeting the COVID-19 case definition and AND any of the following:<br>• Clinical signs indicative of severe systemic illness, respiratory rate ≥30 per minute, heart rate ≥125 beats per minute, SpO2 ≤93% on room air at sea level or PaO2/FIO2 <300 mmHg, OR<br>• Respiratory failure or Acute Respiratory Distress Syndrome (ARDS), (defined as needing high-flow oxygen, non-invasive or mechanical ventilation, or ECMO), evidence of shock (systolic blood pressure <90 mmHg, diastolic BP <60 mmHg or requiring vasopressors), OR<br>• Significant acute renal, hepatic or neurologic dysfunction, OR<br>• Admission to an intensive care unit or death | Participant must have laboratory-confirmed COVID-19 (SARS-CoV-2 RT-PCR-positive symptomatic illness) plus any of the following:<br>• Clinical signs at rest indicative of severe systemic illness (respiratory rate ≥30 breaths per minute, heart rate ≥125 beats per minute, oxygen saturation ≤93% on room air at sea level, or partial pressure of oxygen to fraction of inspired oxygen ratio <300 mmHg)<br>• Respiratory failure (defined as needing high-flow oxygen, noninvasive ventilation, mechanical ventilation or extracorporeal membrane oxygenation)<br>• Evidence of shock (systolic blood pressure <90 mmHg, diastolic blood pressure <60 mmHg, or requiring vasopressors)<br>• Significant acute renal, hepatic, or neurologic dysfunction<br>• Admission to an intensive care unit<br>• Death | A SARS-CoV-2 positive RT-PCR or molecular test result from any available respiratory tract sample (eg, nasal swab sample, sputum sample, throat swab sample, saliva sample) or other sample AND<br>any 1 of the following at any time during the course of observation:<br>• Clinical signs at rest indicative of severe systemic illness (respiratory rate ≥30 breaths/minute, heart rate ≥125 beats/minute, oxygen saturation (SpO2) ≤93% on room air at sea level*, or partial pressure of oxygen/fraction of inspired oxygen (PaO2/FiO2) <300 mmHg) * SpO2 criteria will be adjusted according to altitude per the investigator judgement.<br>• Respiratory failure (defined as needing high-flow oxygen, non-invasive ventilation, mechanical ventilation, or extracorporeal membrane oxygenation [ECMO])<br>• Evidence of shock (defined as systolic blood pressure <90 mmHg, diastolic blood pressure <60 mmHg, or requiring vasopressors)<br>• Significant acute renal, hepatic, or neurologic dysfunction<br>• Admission to the ICU<br>• Death | SARS-CoV-2 RT-PCR positive symptomatic illness with any of the following: clinical signs at rest indicative of severe systemic illness (respiratory rate ≥30 breaths/minute, heart rate ≥125 beats/minute, SpO2 ≤93% on room air at sea level, or PaO2/FiO2 <300 mmHg); respiratory failure (defined as needing high-flow oxygen, non-invasive ventilation, mechanical ventilation, or extracorporeal membrane oxygenation); one or more major organ system dysfunction or failure to be defined by diagnostic testing/clinical syndrome/interventions, including ARDS, acute renal failure, acute hepatic failure, acute right or left heart failure; septic or cardiogenic shock (with shock defined as systolic blood pressure <90 mmHg OR diastolic blood pressure <60 mmHg); acute stroke (ischemic or hemorrhagic), acute thrombotic event (acute myocardial infarction, deep vein thrombosis, pulmonary embolism); requirement for: vasopressors, systemic corticosteroids, or hemodialysis; multisystem inflammatory syndrome in children as per the CDC definition (in participants <21 years), ICU admission; or death. |
| **Primary efficacy endpoint** | First occurrence of COVID-19 starting 14 days after the second dose | First case of SARS-CoV-2 RT-PCR-positive symptomatic illness, in seronegative participants occurring 14 days post second dose | First occurrence of molecularly confirmed, moderate to severe/critical COVID-19, in seronegative participant occurring 14 days after vaccination | First episode of PCR-positive mild, moderate, or severe COVID-19, in seronegative participants occurring 7 days after second dose |

© 2024 Fisher LH et al. *JAMA Network Open.*

| | | | | |
|---|---|---|---|---|
| **Date of COVID-19 onset** | Later date of either:<br>• date of positive PCR test<br>• the date of eligible symptom(s),<br>two dates should be within 14 days of each other | Earliest collection date of positive central lab RT-PCR or local lab RT-PCR. | Date when any sign(s) or symptom(s) suggesting possible COVID-19 | Minimum date of the following events (both events must occur):<br>• date of PCR positive result from the UWVL<br>• date of the start of mild, moderate, or severe COVID-19 disease from the COVID-19 Endpoint assessment CRF. |
| **COVID-19 Symptoms Triggering Illness Visits** | Participants instructed to arrange an Illness Visit with site to collect an NP swab within 72 hours of:<br>• Fever (temperature ≥38ºC) or chills; shortness of breath or difficulty breathing; or cough for any duration, OR<br>• Fatigue, muscle or body aches, headache, new loss of taste or smell, sore throat, congestion or runny nose, nausea or vomiting, or diarrhea lasting at least 48 hours | Participant instructed to initiation Illness Visits for PCR testing if they experienced:<br>• Fever, shortness of breath, or difficulty breath for any duration, OR<br>• Chills, cough, fatigue, muscle aches, body aches, headache, new loss of taste, new loss of smell, sore throat, congestion, runny nose, nausea, vomiting, or diarrhea present for at least 2 days | The triggers to proceed with home-collection of the nasal swabs on COVID-19 Day 1-2 and to proceed with the COVID-19 Day 3-5 visit were prespecified as follows:<br>• A positive RT-PCR result for SARS-CoV-2, through a private or public laboratory<br>independent of the study, whether symptomatic or asymptomatic<br>OR<br>• New onset or worsening of any 1 of the symptoms, which lasts for at least 24 hours, not otherwise explained: headache; malaise (appetite loss, generally unwell, fatigue, physical weakness); myalgia (muscle pain); chest congestion; cough; runny nose; shortness of breath or difficulty breathing (resting or on exertion); sore throat; wheezing; eye irritation or discharge; chills; fever (≥38.0°C or ≥100.4°F); pulse oximetry value ≤95%,which is a decrease from baseline; heart rate ≥90 beats/minute at rest, which is an increase from baseline; gastrointestinal symptoms (diarrhea, vomiting, nausea, abdominal pain); neurologic symptoms (numbness, difficulty forming or understanding speech); red or bruised looking toes; skin rash; taste loss or new/changing sense of smell; symptoms of blood clots: | Participant was directed via the eDiary to begin daily nasal self-swabbing for PCR testing at home for a total of 3 days if they experienced:<br>• Fever (temperature ≥38ºC) or chills for any duration, OR<br>• Two consecutive days of: new onset or worsening of cough compared with baseline; new onset or worsening of shortness of breath or difficulty breathing over baseline; new onset fatigue; new onset generalized muscle or body aches; new onset headache; new loss of taste or smell; acute onset sore throat; acute onset congestion or runny nose; new onset nausea or vomiting; or new onset of diarrhea |

© 2024 Fisher LH et al. *JAMA Network Open.*

| | | | | |
|---|---|---|---|---|
| | | | pain/cramping, swelling or redness in your legs/calves; confusion; bluish lips or face; or clinical suspicion/judgement by investigator of symptoms suggestive for COVID-19 | |

In this analysis, we used the same harmonized COVID-19 comorbidity and SARS-CoV-2 exposure risk definitions defined in Theodore et al.[1] For convenience, these definitions are reproduced (with author permission) below:

**Baseline Comorbidities**

In this analysis, comorbid conditions (yes or no) were defined as the presence of a given medical condition indicated in either the medical history eCRF or the comorbid questionnaire CRF data. Comorbid conditions listed by CDC as associated with severe COVID-19 were first mapped to MedDRA coding. Specifically, the CDC updated on Feb 15, 2022, the listing of underlying medical conditions associated with higher risk for severe COVID-19 (https://www.cdc.gov/coronavirus/2019-ncov/science/science-briefs/underlying-evidence-table.html). All of the medical conditions in this CDC list were mapped to MedDRA 24.0- English version coding for the applicable Preferred Term (PT) and High-level Term (HLT) for each medical condition. The medical diagnosis listed in medical history eCRF of each participant were available as MedDRA terms. The frequencies of occurrence of each of these conditions were tabulated and included as an independent variable. The medical conditions and subcategories with a frequency of occurrence <5% were combined or eliminated for the construction of variables analyzed in the final dataset (Supplementary Table 4 from Theodore et al.) The mapped coding was then used to determine the presence of each condition as listed in the medical history eCRF.

**Occupational Risk**

Occupational risk was determined by attributing Occupational Safety and Health Administration (OSHA) hazard recognition scores to self-reported workplace information provided by participants. OSHA functions as a regulatory agency under the United States Department of Labor to ensure safe and healthful working conditions, and as such, defined categories in response to the Covid-19 pandemic to aid in the assessment and mitigation of exposure risk in the workplace. Low exposure risk jobs have minimal contact with the public or coworkers. Medium exposure risk jobs have frequent or sustained close contact with the public or coworkers in outdoor or well-ventilated settings. High exposure risk jobs include close or poorly ventilated working conditions with known or suspected sources of SARS-CoV-2 (such as a hospital, grocery store or public transit). Very high exposure risk jobs are performing specific medical, postmortem or laboratory procedures. If individuals selected more than one category, the maximum score was taken. High and very high exposure risk categories were combined for analysis.

**Living Situation Risk**

Living situation risk synthesizes variables across all four trials and is scored on a scale of low, medium, high, or very high risk. It is based on housing type for the Moderna trial and number of co-habitants for the other 3 trials. For the AstraZeneca, Janssen, and Novavax trials, low, medium, high, and very high risk conditions corresponded to 0-1, 2, 3 and 4 or more co-habitants, respectively. For the Moderna study, individuals self-reported the housing type(s) that applied. Each housing type was assigned to low (participants specified as not having risk of exposure related to housing), medium (single-family or detached housing, housing without shared entrances or elevators), high (congregate settings such as dormitory, group housing, or high density such as apartments with shared entrances or elevators), or very high (nursing homes, long-term care facilities, shelters, and multi-family dwellings) risk categories. If participants selected more than one housing type, the highest risk score was taken. If “other” was selected, a value was imputed using the most frequent category within a given study.

© 2024 Fisher LH et al. *JAMA Network Open.*

**Protocol-specific schedule of illness visits, specimen types, and viral load quantitation**

Although COVID-19 endpoint definitions were harmonized across the four trials, the schedule of illness visits, and specimen types varied by parent protocol. Moderna collected a nasal/nasopharyngeal (NP) swab on illness visit 1, followed by saliva on illness visit days 3, 5, 7, 9, 14, 21, and 28. AstraZeneca collected nasal/NP swabs on illness visit days 1, 14, 21, and 28; and saliva on days 1, 3, 5, 8, 11, 14, 21, and 28. Janssen collected nasal/NP swabs on illness visit 1, and every other illness visit day through day 23. Novavax collected nasal/NP swabs only from illness visit days 1, 2, and 3 (**eFigure 1**).

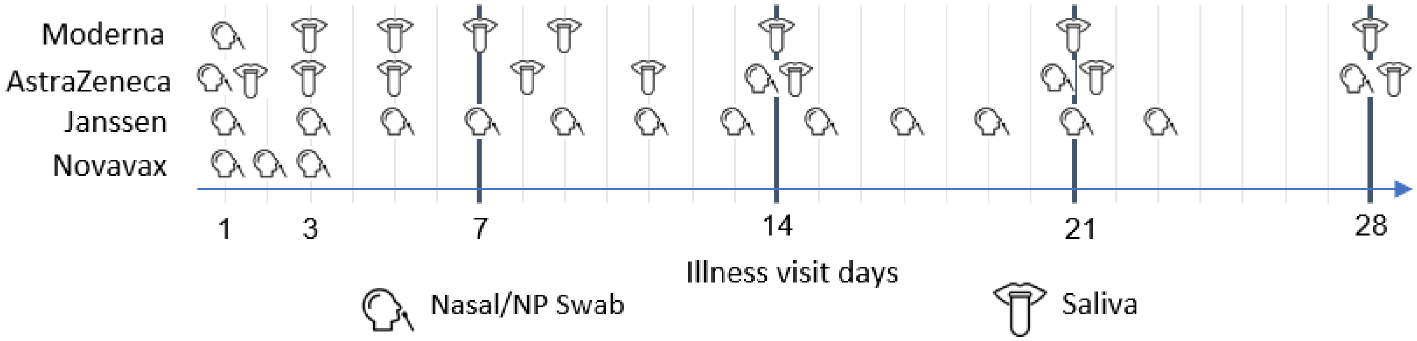


**eFigure 1. Summary of protocol-defined sampling collection for illness visits, triggered by symptom onset.** Nasal and nasopharyngeal (NP) swabs are denoted by a head with swab, and saliva specimens are denoted by a mouth with a tube.

Specimens collected from participants enrolled in the Moderna trial were quantified by Eurofins Viracor.[2] The RT-PCR assay targets two genes (N1 and N2) in a single channel and conversion to a standardized viral load has been previously described.[3] Specimens collects from participants of the AstraZeneca trial were quantified at LabCorp using an RT-PCR assay targeting the E and ORF1ab genes separately. University of Washington Virology (UWVL) quantified specimens from both Janssen and Novavax using the Abbott m-2000 SARS-CoV-2 real-time RT-PCR, which targets the N1 and N2 genes in a single channel.[4] For specimens from the Janssen trial, swabs were first tested at a local lab and remnants of locally positive swabs were sent to University of Washington for quantification and sequencing (if selected). Because of the international nature of this trial, not all study sites had access to suitable local PCR testing capacity. If local testing was not available, specimens were shipped to Covance/Labcorp for qualitative PCR testing, and presumptive positive specimens were then sent to University of Washington for quantification. As a result, swabs that were not tested locally underwent an additional freeze-thaw cycle before quantitation at University of Washington.

**Urchin – A Tool for Predicting SARS-CoV-2 Variants from Spike Sequences**

The gold-standard approach to determine the lineage (and hence WHO variant status) of a SARS-CoV-2 sequence is with either the PANGOLIN or NextClade software tools. Because these tools provide information about the specific lineage of a given virus, they require a whole-genome sequence as input. These studies focused on the spike protein, and as such, sequences for some studies, for various reasons, were only available as the S gene (nucleotide sequence) or the spike protein (protein sequence). We needed a way to determine the WHO-defined variant label for these spike-only sequences.

We accomplished this with a predictive model, which we implemented as a Shiny[5]-based web tool named Urchin. Urchin uses an optimized learner for predicting the WHO Greek-lettered variant name of a given SARS-CoV-2 spike protein sequence. In this particular analysis, Urchin's predictions were used to determine the variant labels for sequences from the Moderna (n = 790) and AstraZeneca (n = 680) studies.

To train and validate the Urchin model we started with a corpus of approximately 1.93 million sequences obtained from the GISAID database[6] on August 21, 2021 (EPI-SET doi 10.55876/gis8.230822ky). We later updated our data set to account for emergence of the Omicron variants, specifically 10,000 sequences of both BA.1 and BA.2 along with the 239 BA.3 sequences available at the date of retrieval (March 20, 2022). These sequences were aligned using the MAFFT multiple sequence alignment program,[7] with manual touch-ups as needed. From here, we generated a feature set by rendering the sequence data into a set of binary indicator variables for each position, leading to a feature for each observed amino acid at every site (e.g., "position 614 is 'G'"), excluding gaps ("-") and unknown amino acids ("X"). As a dimensionality reduction step, features that were within 20 instances of being perfectly homogenous were screened out. To ensure that signature sites characteristic of variant definitions would not be accidentally filtered out, we identified a set of signature sites for all extant variants and prespecified them to be included as training features, regardless of whether they would pass the dimensionality reduction filter. These signature sites are enumerated in **eTable 2**. This dimensionality reduction step reduced the derivation set from 17,488 features down to 9,467 features. We then conducted a 70:30 training:validation split of the

© 2024 Fisher LH et al. *JAMA Network Open.*

data, using random selection and stratifying by variant. This resulted in a training set of 1,348,494 sequences and a validation set of 577,927 sequences.

**eTable 2. Variant-specific signature sites that were included in the homogeneity screening process (all positions indexed to the NC_045512 reference strain)[8]**

| WHO Label | Spike Position |
|---|---|
| Alpha | 501, 570, 614, 681, 716, 982, 1118 |
| Beta | 80, 215, 417, 484, 501 ,614, 701 |
| Gamma | 18, 20, 26, 138, 190, 417, 484, 501, 614, 655, 1027, 1176 |
| Delta | 19, 95, 142, 158, 452, 478, 614, 681, 950 |
| Iota | 5, 95, 253, 484, 614, 701 |
| Eta | 52, 67, 484, 614, 677, 888 |
| Kappa | 95, 142, 154, 452, 484, 614, 681, 1071 |
| Lambda | 75, 76, 246, 452, 490, 614, 859 |
| Mu | 95, 143, 144, 145, 346, 484, 501, 614, 681, 950 |
| Theta | 265, 484, 501, 614, 681, 1092, 1101, 1176 |
| Zeta | 484, 614, 1176 |
| Epsilon | 13, 152, 452, 614 |

To optimize our model and eliminate any bias that might occur from variants that were over-represented in the database, we created an exploration set from the training set by randomly sampling N sequences for each variant, where N is equal to 38, the frequency of the least-represented variant in the training data (Theta). Since the training data contained sequences for 15 variants (including the Ancestral lineage), this resulted in an exploration set containing 570 sequences. This exploration set was then used as the training set for an initial learner.

We approached this as a multi-class problem, to develop a learner that would predict the Greek-lettered variant of any sequence, as provided by GISAID's metadata file. All sequences from the A.1 Ancestral lineage and the B.1 basal outbreak lineage (with the G614G mutation) were grouped into a single "Ancestral Strain" variant category. For our learning method, due to the discrete nature of mutations and their associations with variants, we selected extreme gradient boosting (XGBoost[9]) with the multiclass log loss evaluation metric and 50 rounds of boosting iterations.

We performed 5-fold cross validation to estimate the error with the exploration set. This resulted in a cross-validated predictive accuracy (the proportion of correct predictions) of 0.988 (95% CI: 0.975, 0.995). Using the same hyperparameters, we trained a single model to predict the variant of the validation set, resulting in a predictive accuracy of 0.993 (0.9927, 0.9931). Only 79 of the total 9467 features held predictive importance in this model and thus were used to define our new feature set. These final features (79 residues across 60 unique positions) are enumerated in **eTable 3**.

**eTable 3. The final set of features (79 features across 60 unique positions) used by the final predictive model (all positions indexed to the NC_045512 reference strain).**

| Spike Position | Amino Acid |
|---|---|
| 13 | I, S |
| 19 | R, T, I |
| 20 | N |
| 24 | L |
| 52 | R, Q |

| | |
|---|---|
| 63 | T |
| 68 | I |
| 69 | H |
| 70 | V |
| 71 | S |
| 72 | G |
| 75 | V, G |
| 80 | A, D |
| 95 | I, T |
| 98 | S |
| 126 | V |
| 142 | G |
| 143 | V |
| 144 | S, Y |
| 145 | N |
| 150 | K |
| 152 | C, W |
| 153 | M |
| 190 | R |
| 213 | G |
| 215 | Y, D |
| 222 | A |
| 242 | L |
| 253 | G |
| 258 | W |
| 259 | T |
| 261 | G |
| 262 | A |
| 264 | A |
| 265 | Y |
| 271 | Q |
| 272 | P |
| 275 | F |
| 323 | T |
| 346 | K, R |
| 371 | L |
| 405 | N |
| 417 | N |
| 452 | L, R |
| 477 | S |
| 484 | K, E |
| 490 | F |

© 2024 Fisher LH et al. *JAMA Network Open.*

| | |
|---|---|
| 496 | G |
| 501 | N, Y |
| 570 | D |
| 677 | H |
| 681 | P, H |
| 701 | A, V |
| 859 | T, N |
| 888 | F, L |
| 950 | N |
| 966 | L |
| 969 | N |
| 1101 | Y |
| 1176 | F, V |

Using this refined set of features, we trained a final model on the full training set, using the same XGBoost parameters as before. Validating this model on the holdout set of 577,927 sequences, it performed with a predictive accuracy of 0.9929 (0.9927, 0.9931). This is the final model that was selected for use with the Urchin web tool.

The results across both studies then underwent a phylogenetic analysis in order to investigate any potential miscalls from Urchin. Both sequence sets were combined and phylogenetic trees were generated using the PhyML software with the Blosum62 model. From these trees, 20 sequences predicted to be of the Ancestral Strain by Urchin appeared to be miscalls, and this was confirmed with a tree built from a larger set of sequences. The 20 miscalls were confirmed to be: 9 Lambda, 5 Iota, 4 Beta, and 2 which were either Gamma or Zeta. Of these 20 miscalls, 6 were from the Moderna study (0.7% miscalls) and 14 from AstraZeneca (2% miscalls). The miscalls were due to either missing sequence content, rare mutations, or, in the case of Lambda, a 13-AA deletion covering spike positions 64 through 76 that is inconsistently found in Lambda and was confusing our learner by defeating the characteristic G75V mutation.

This predictor is available online as the Urchin web tool, which can be accessed at https://urchin.fredhutch.org/. Users can submit a set of spike sequence data in FASTA format and Urchin will return the predicted WHO variant labels. The sequences do not need to be aligned, and they are each transformed into the 79 features required by the learner. The final model uses these features to predict the variant label for each submitted sequence. The results are reported back to the user and can be downloaded as a CSV file. Urchin is available to the public for the sake of reproducibility, https://urchin.fredhutch.org/ as it has not been retrained for recent variants (particularly emerging Omicron subvariants and recombinants), so it will be of limited use with modern sequences. The source code is available at https://github.com/jamesprg/urchin.

**Multiple imputation of SARS-CoV-2 Variants**

While all protocols prioritized obtaining a sequence from each infection observed in the trial, successful sequencing was not always possible. There are several reasons why it was not possible to obtain a viral sequence, even from repeated sampling over the course of an infection. In general, sequencing was not even attempted if the specimen was found to have a low viral load (or high cycle threshold), although the specific sequencing thresholds will vary by lab. Sequencing could also be missing for other reasons, including low sample volume or poor specimen quality. As a result, sequencing was not available for 20.6% of the analysis cohort and were classified as such for the primary analyses.

The extensive genomic data that was collected, sequenced, and openly shared from specimens worldwide through GISAID provided an opportunity for imputation analyses to fill in those missing variants. As the specimen level, the information is highly variable in the GISAID metadata; as such, we focused on the strongest predictors of circulating viruses: space and time.

We used GISAID metadata of specimens from individuals in the 8 countries represented in our analysis population: Argentina, Brazil, Chile, Colombia, Mexico, Peru, South Africa, and the United States of America. The specimens in our analysis cohort were classified as one of the WHO-named variants (Alpha, Beta, Delta, Gamma, etc.) or Other. Since this analysis was limited to the early part of the pandemic, the Other category was primarily composed of Ancestral lineages, but had other minor lineages that did not reach the level of a named variant. We excluded specimens with impossible variant-date combinations, such as Delta in November of 2020.

© 2024 Fisher LH et al. *JAMA Network Open.*

For imputation analyses, we supplemented the analysis data with the estimated proportion of circulating variants at the time of infection, based on the local GISAID metadata. In particular, for each infection in the analysis dataset, we estimate the proportion of cases attributed to each variant by summarizing a two-week window around the date of COVID-19 onset in local GISAID data. For swabs collected from participants within the United States, state-level GISAID data was used, while country-level GISAID data was used for all other infections. **eFigure 2** provides a visual representation of the available data used to estimate the distribution of circulating variants by infection in Chile. In the example, we would attribute all of the circulating cases to the Other category for the first infection (left-most red vertical line); in contrast, for the last infection missing a variant (the rightmost vertical line), the estimated distribution of variants at that time would inclue nearly all of the observed variants (except Delta), to varying degrees.

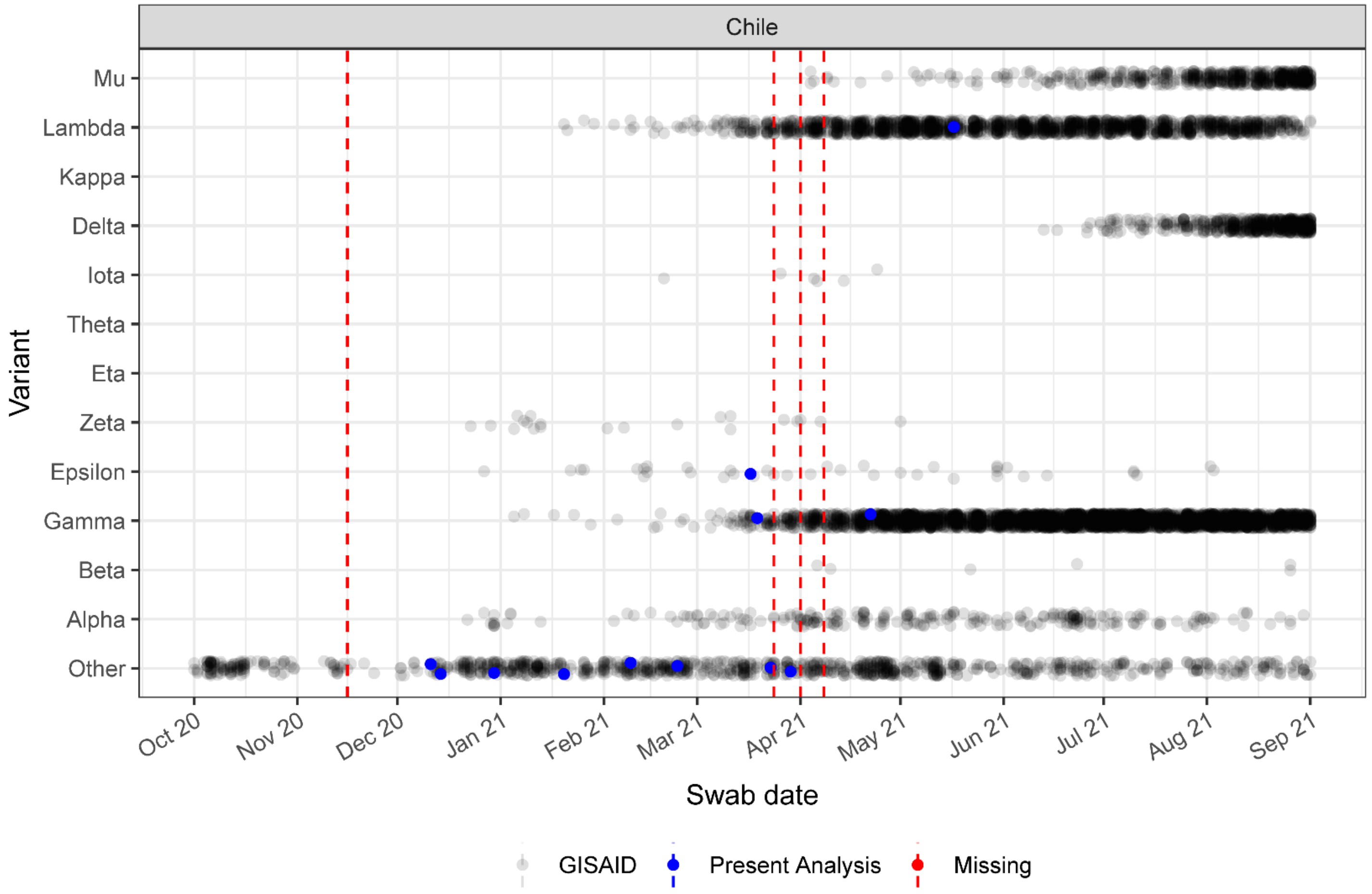


**eFigure 2. Example of data used for imputation analyses.** Gray dots represent the observed variants sequencing in Chile over time in the GISAID database, blue dots indicate samples from the analysis dataset that have successfully been sequenced, and the red dashed lines denote the dates of illness visit day 1 swabs that are missing sequencing information. Other includes Ancestral strain, as well as all other non-WHO-named lineages.

We used these estimated proportions of circulating variants at the time of infection to do a simple imputation analysis. Specifically, we imputed the missing variant by taking a random draw from the multinomial distribution, with variant probabilities defined by estimated proportions of circulating variants at the time of infection and fitting the multivariate regression model (**Figure 4**). Regression results over 20 imputed datasets were then combined and summarized to account for the additional variability using the mice package in R.[10] Global nominal p-values were defined as the median of the multiple p-values obtained from Wald tests for each imputed dataset only when standard approaches failed.[11]

**Sensitivity and Exploratory analyses**

© 2024 Fisher LH et al. *JAMA Network Open.*

We consider several sensitivity and exploratory analyses. To examine the robustness of our conclusions to the variant imputation analysis, we repeated the multivariate model on the subset of participants with successful sequencing. Two analyses explored the sensitivity of our conclusions to the variant identification, by restricting the multivariate analysis to the subset of those identified as being infected by the Ancestral variant only and by using Hamming distances in lieu of variant calls in the subset of participants with successful sequencing. To explore the sensitivity of our conclusions to the inclusions of negative PCR results, we repeated the multivariate analysis restricting to those with detectable viral load. The sensitivity analysis restricted to those enrolled in the US examined the impact of including international trial sites, primarily from the Janssen trial, on the primary conclusions. The sensitivity analysis limited to those enrolled in the Janssen trial explored the sensitivity of our conclusions within the largest trial. As an exploratory analysis, the multivariate model was fit including country-specific smoothed calendar time trends, to allow for potential confounding of viral load by local epidemic dynamics. In particular, the GAM model is an extension of the multivariate linear regression used in the primary analysis that flexibly models non-linear local temporal trends in COVID-19 incidence using cubic regression splines.

**Severity prediction**

In a post-hoc exploratory analysis, we examined the utility of log viral load at diagnosis in the prediction of severe COVID-19. This was only feasible for the Janssen trial, where two measures of viral load were considered: the log viral load at diagnosis (i.e., first illness associated swab) and the area under the longitudinal log viral load curve (VL-AUC), estimated using the trapezoidal rule over 28 days. Additional predictors included a full set of baseline participant characteristics and infection characteristics, summarized below:

| **Variable sets** | **Variables** |
|---|---|
| Baseline demographics | Age (continuous), sex, country, race, ethnicity, BMI at baseline (continuous), risk of COVID-19 exposure (categorical), living conditions (categorical) |
| Baseline comorbidities (binary) | Lung disease, cardiovascular disease, obesity, diabetes, liver disease, HIV, history of smoking, asthma, hypertension, COVID-19 comorbidities |
| Infection characteristics | Infecting variant, initial VL, AUC of VL trajectory (VL-AUC), days since onset VL measurements began |

Superlearner modeling, using the negative log-likelihood loss function, and a library of adaptive and non-adaptive learners and classifiers, was employed. Cross-validation was performed at two levels: five-fold outer level to compute the cross-validated area under the ROC curve (CV-AUC), and 5-fold inner level to estimate ensemble weights. CV-AUC and influence curve-based confidence intervals were computed for the ensemble model (Superlearner), discrete Superlearner, and the individual learners.[12] Marginal and conditional variable importance were assessed using the vimp package in R.[13]

For predictive models that included a single covariate (either log VL at first illness associated PCR test, or VL-AUC), learner libraries included glm and gam (SL.mean, SL.glm, SL.gam). Predictive models adjusting for additional baseline covariates used a larger collection of learner libraries that also included glm interactions (SL.glm.interaction), elastic net (SL.glmnet; alpha=0, 0.25, 0.5, 0.75, 1 ), random forests (SL.ranger), and gradient-boosted machines (SL.xgboost).

© 2024 Fisher LH et al. *JAMA Network Open.*

## Supplemental Results

### eTable 1. Additional baseline characteristics

| | Moderna (N=594) | AstraZeneca (N=97) | Janssen (N=916) | Novavax (N=60) | Total (N=1667) |
|---|---|---|---|---|---|
| **Age at enrollment (years)** | | | | | |
| Mean (SD) | 48.1 (14.4) | 46.3 (14.9) | 46.1 (14.9) | 41.8 (14.9) | 46.7 (14.7) |
| **Underrepresented Minority (US Only)** | | | | | |
| Yes | 165 (27.8) | 15 (15.5) | 68 (7.4) | 12 (20.0) | 260 (15.6) |
| No | 429 (72.2) | 55 (56.7) | 207 (22.6) | 44 (73.3) | 735 (44.1) |
| **BMI at Baseline Visit ($kg/m^2$)** | | | | | |
| Mean (SD) | 30.4 (7.0) | 29.7 (6.3) | 28.1 (5.6) | 29.3 (7.0) | 29.0 (6.3) |
| **Baseline comorbidities: n (%)** | | | | | |
| Lung Disease | 24 (4.0) | 3 (3.1) | 52 (5.7) | 8 (13.3) | 87 (5.2) |
| Cardiovascular Disease | 153 (25.8) | 27 (27.8) | 194 (21.2) | 14 (23.3) | 388 (23.3) |
| Obesity | 258 (43.4) | 46 (47.4) | 257 (28.1) | 27 (45.0) | 588 (35.3) |
| Diabetes | 60 (10.1) | 9 (9.3) | 65 (7.1) | 6 (10.0) | 140 (8.4) |
| Kidney Disease | 4 (0.7) | 0 (0.0) | 5 (0.5) | 0 (0.0) | 9 (0.5) |
| Liver Disease | 5 (0.8) | 3 (3.1) | 7 (0.8) | 0 (0.0) | 15 (0.9) |
| HIV | 3 (0.5) | 2 (2.1) | 14 (1.5) | - | 19 (1.1) |
| History of Smoking | 9 (1.5) | 17 (17.5) | 11 (1.2) | 18 (30.0) | 55 (3.3) |
| Asthma | 58 (9.8) | 3 (3.1) | 49 (5.3) | 6 (10.0) | 116 (7.0) |
| Hypertension | 148 (24.9) | 26 (26.8) | 184 (20.1) | 13 (21.7) | 371 (22.3) |
| **Risk of Exposure to SARS-CoV-2 as per OSHA (Imputed): n (%)** | | | | | |
| Lower Exposure Risk | - | 30 (30.9) | 848 (92.6) | 34 (56.7) | 912 (54.7) |
| Medium Exposure Risk | 150 (25.3) | 45 (46.4) | 20 (2.2) | 19 (31.7) | 234 (14.0) |
| High Exposure Risk | 444 (74.7) | 22 (22.7) | 48 (5.2) | 7 (11.7) | 521 (31.3) |
| **Housing Type: n (%)** | | | | | |
| Low-Risk Housing | 94 (15.8) | - | - | - | 94 (5.6) |
| Medium-Risk Housing | 450 (75.8) | 71 (73.2) | 534 (58.3) | 60 (100.0) | 1115 (66.9) |
| High-Risk Housing | 30 (5.1) | 26 (26.8) | 3 (0.3) | - | 59 (3.5) |
| Very-High-Risk Housing | 20 (3.4) | - | 379 (41.4) | - | 399 (23.9) |
| **Living Condition: n (%)** | | | | | |
| Low-Risk Living Condition | 94 (15.8) | 33 (34.0) | 329 (35.9) | 45 (75.0) | 501 (30.1) |
| Medium-Risk Living Condition | 450 (75.8) | 16 (16.5) | 305 (33.3) | 11 (18.3) | 782 (46.9) |
| High Risk-Living Condition | 30 (5.1) | 19 (19.6) | 206 (22.5) | 2 (3.3) | 257 (15.4) |
| Very-High-Risk Living Condition | 20 (3.4) | 29 (29.9) | 76 (8.3) | 2 (3.3) | 127 (7.6) |

© 2024 Fisher LH et al. *JAMA Network Open.*

## Summary of univariate model results

Viral load at diagnosis was highly variable, with a median viral load of 6.18 $log_{10}$ copies/mL (interquartile range 4.66-7.12 $log_{10}$ copies/mL). Among the three protocols that provided data that included undetectable viral loads, 6.3% (68/1073) of participants met this criterion. Distributions of $log_{10}$ viral load and univariate analyses of factors associated with viral load at diagnosis are summarized in **Figure 3** and **eTable 2**.

Parent protocol was univariately associated with viral load (adjusted p<0.01), with placebo cases in Janssen having an estimated 0.83 $log_{10}$ copies/mL lower mean viral load relative to those in the reference Moderna protocol (95% CI: 1.04 to 0.62 lower) (**Figure 3A, eTable 2**). Additionally, country was associated with viral load: Colombia and South Africa had significantly lower mean viral loads compared to the reference US. Given that 95% of non-US cases were enrolled in the Janssen trial, these associations between country and protocol and viral load are likely related.

Other baseline factors univariately associated with viral load at diagnosis included participant race, having one or more comorbidities, and SARS-CoV-2 exposure risk (**eTable 2**). Similar viral load distributions were observed among participants who had severe disease vs. those with non-severe disease (**Figure 3B**).

Viral load distributions were highest closest to COVID-19 onset (**Figure 3D, eTable 2**), with an estimated 0.26 $log_{10}$ copies/mL lower mean viral load each additional day (95% CI: 0.34 to 0.18 lower; adjusted p<0.01).

Additionally, there were apparent differences in viral load among the SARS-CoV-2 variants (adjusted p<0.01). Viral loads corresponding to infections missing sequences were lower than those with sequences, with median viral loads of 3.62 and 6.48 $log_{10}$ copies/mL, respectively (**Figure 3C**). This may be due to an inherent threshold for successful sequencing. The univariate analysis estimated Beta, Gamma, and Mu to have between 0.5 and 1.2 $log_{10}$ copies/mL lower and Delta to have 0.28 $log_{10}$ copies/mL higher mean viral load at diagnosis relative to Ancestral. However, there were just seven Delta infections in this cohort. In these univariate analyses, infecting variant explained approximately 23% of the variability in $log_{10}$ viral load, although this was primarily attributable to the difference in viral load between individuals with and without sequences.

Thus, while several factors were found to be associated with viral load at diagnosis based on univariate analyses, none of the participant characteristics, beyond infecting variant, explained more than 3.7% of the observed variability.

**eTable 2. Univariate linear regression results for placebo infections.** Estimated mean difference in $log_{10}$ viral load (VL) at diagnosis between each covariate category and the reference category, or per unit increase in the covariate in the case of continuous covariates, along with 95% confidence interval and nominal p-value, based on univariate linear regression models. Global nominal p-values from Wald Test to test coefficients for categorical variables with > 2 levels. Adjusted p-values account for multiplicity and are corrected using the Holm method. Adjusted $R^2$ are included from the univariate model fit.

| Covariate | Mean Difference $log_{10}$(VL) (95% CI) | Nominal P-value | Global Nominal P | Adjusted P-value | Adjusted $R^2$ |
|---|---|---|---|---|---|
| Age Category (Ref: 18-29) | | | | | |
| 30-39 | 0.15 (-0.20, 0.50) | 0.40 | 0.42 | 1.00 | 0.000 |
| 40-49 | 0.12 (-0.19, 0.44) | 0.44 | | | |
| 50-64 | 0.25 (-0.04, 0.55) | 0.09 | | | |
| 65+ | 0.30 (-0.06, 0.67) | 0.11 | | | |
| Sex (Ref: Female) | | | | | |
| Male | 0.08 (-0.12, 0.27) | 0.44 | | 1.00 | 0.000 |
| Race (Ref: White) | | | | | |
| American Indian or Alaska Native | -0.56 (-0.87, -0.25) | <0.01 | <0.01 | <0.01 | 0.025 |
| Asian | -0.16 (-0.79, 0.47) | 0.62 | | | |
| Black or African American | -0.96 (-1.31, -0.62) | <0.01 | | | |
| Multiple | -0.29 (-0.68, 0.11) | 0.16 | | | |
| Other | 1.40 (0.29, 2.50) | 0.01 | | | |
| Not Reported | -0.52 (-1.12, 0.08) | 0.09 | | | |
| Ethnicity (Ref: Not Hispanic or Latino) | | | | | |
| Hispanic or Latino | -0.29 (-0.48, -0.09) | <0.01 | 0.01 | 0.19 | 0.004 |

© 2024 Fisher LH et al. *JAMA Network Open.*

| | | | | | |
|---|---|---|---|---|---|
| Not Reported | -0.56 (-1.46, 0.35) | 0.23 | | | |
| BMI at Baseline Visit | 0.00 (-0.01, 0.02) | 0.70 | | 1.00 | 0.000 |
| BMI Category (Ref: Healthy weight) | | | | | |
| Underweight | -0.58 (-1.86, 0.69) | 0.37 | 0.65 | 1.00 | 0.000 |
| Overweight or obese | 0.01 (-0.21, 0.23) | 0.93 | | | |
| Infecting Variant (Ref: Ancestral) | | | | | |
| Alpha | -0.54 (-0.98, -0.10) | 0.015 | <0.01 | <0.01 | 0.029 |
| Beta | -1.24 (-1.70, -0.77) | <0.01 | | | |
| Gamma | -0.49 (-0.82, -0.17) | 0.003 | | | |
| Epsilon | -0.08 (-0.62, 0.46) | 0.767 | | | |
| Zeta | -0.21 (-0.59, 0.18) | 0.287 | | | |
| Iota | 0.03 (-1.29, 1.34) | 0.968 | | | |
| Delta | 0.28 (-0.94, 1.50) | 0.654 | | | |
| Lambda | -0.26 (-0.69, 0.18) | 0.248 | | | |
| Mu | -0.86 (-1.31, -0.40) | <0.01 | | | |
| Symptom Severity (Ref: Not Severe) | | | | | |
| Severe | -0.14 (-0.41, 0.13) | 0.31 | | 1.00 | 0.000 |
| Days since COVID-19 Onset | -0.26 (-0.34, -0.18) | <0.01 | | <0.01 | 0.025 |
| COVID-19 Comorbidities | -0.31 (-0.52, -0.11) | <0.01 | | 0.05 | 0.005 |
| Parent Protocol (Ref: Moderna) | | | | | |
| AstraZeneca | -0.02 (-0.45, 0.41) | 0.94 | <0.01 | <0.01 | 0.037 |
| Janssen | -0.83 (-1.04, -0.62) | <0.01 | | | |
| Novavax | -0.46 (-0.99, 0.07) | 0.09 | | | |
| Country (Ref: USA) | | | | | |
| Argentina | -0.29 (-0.71, 0.14) | 0.18 | <0.01 | <0.01 | 0.028 |
| Brazil | -0.16 (-0.49, 0.16) | 0.33 | | | |
| Chile | -0.11 (-1.10, 0.88) | 0.83 | | | |
| Colombia | -0.82 (-1.13, -0.51) | <0.01 | | | |
| Mexico | -0.89 (-2.04, 0.25) | 0.13 | | | |
| Peru | -0.18 (-0.59, 0.23) | 0.39 | | | |
| South Africa | -1.28 (-1.73, -0.83) | <0.01 | | | |
| US Underrepresented Minority | -0.44 (-0.73, -0.16) | <0.01 | | 0.04 | 0.025 |
| Lung Disease (Ref: No) | -0.34 (-0.78, 0.10) | 0.13 | | 1.00 | 0.001 |
| Cardiovascular Disease (Ref: No) | 0.13 (-0.10, 0.36) | 0.26 | | 1.00 | 0.000 |
| Obesity (Ref: No) | 0.04 (-0.16, 0.25) | 0.68 | | 1.00 | 0.000 |
| Diabetes (Ref: No) | 0.21 (-0.14, 0.56) | 0.25 | | 1.00 | 0.000 |
| Kidney Disease (Ref: No) | 0.15 (-1.19, 1.49) | 0.83 | | 1.00 | 0.000 |
| Liver Disease (Ref: No) | 0.36 (-0.67, 1.40) | 0.49 | | 1.00 | 0.000 |
| HIV (Ref: No) | -0.32 (-1.24, 0.60) | 0.50 | | 1.00 | 0.000 |
| History of Smoking (Ref: No) | 0.08 (-0.47, 0.63) | 0.77 | | 1.00 | 0.000 |
| Asthma (Ref: No) | -0.32 (-0.70, 0.07) | 0.10 | | 1.00 | 0.001 |
| Hypertension (Ref: No) | 0.14 (-0.10, 0.37) | 0.25 | | 1.00 | 0.000 |
| Risk of Exposure to SARS-CoV-2 as per OSHA (Ref: Lower Exposure Risk) | | | | | |
| Medium Exposure Risk | 0.61 (0.32, 0.90) | <0.01 | <0.01 | <0.01 | 0.028 |
| High Exposure Risk | 0.74 (0.52, 0.95) | <0.01 | | | |
| Living Condition (Ref: Low Risk Living Condition) | | | | | |
| Low Risk Living Condition | 0.10 (-0.20, 0.41) | 0.51 | 0.03 | 0.49 | 0.0035 |
| Medium Risk Living Condition | 0.32 (0.04, 0.61) | 0.03 | | | |
| Very High Risk Living Condition | -0.09 (-0.52, 0.34) | 0.68 | | | |

**Summary of univariate sensitivity analyses**

© 2024 Fisher LH et al. *JAMA Network Open.*

As a brief sensitivity analysis, we compared three univariate analyses of infecting variant (**eTable 3**). Overall, all three approaches provided similar estimates of mean difference in VL relative to the Ancestral variant. Estimates from imputation are comparable to those obtained in both the complete case and observed data analyses. All confidence intervals overlap, although there are some small differences in individual variant estimates. The most notable difference in estimates was seen in among Gamma infections, where the complete case analysis estimated the difference in mean VL at 0.49 $\log_{10}$ copies/mL lower than Ancestral (95% CI: 0.82 to 0.17 lower) and the imputation analysis estimated Gamma infections to be 0.22 $\log_{10}$ copies/mL lower compared to Ancestral (95% CI: 0.63 lower to 0.20 higher).

© 2024 Fisher LH et al. *JAMA Network Open.*

**eTable 3. Comparison of model results from three univariate analysis of infecting variant.** The observed data approach, which classifies missing variants as such; the complete case analysis, which limits the univariate analysis to the subset with successful sequencing; and the multiple imputation univariate analysis, which imputes missing variants based on the observed distribution of circulating variants near the swab collection, are compared. Multiple imputation results combine 20 imputations.

| | Observed Data (N = 1,667) | | Successfully Sequenced (N=1,323) | | Multiple Imputation (N = 1,667) | |
|---|---|---|---|---|---|---|
| **Infecting Variant Relative to Ancestral (N observed)** | **Mean Difference $\log_{10}$(VL) (95% CI)** | **Nominal P-value** | **Mean Difference $\log_{10}$(VL) (95% CI)** | **Nominal P-value** | **Mean Difference $\log_{10}$(VL) (95% CI)** | **Nominal P-value** |
| **Alpha (58)** | -0.54 (-1.02, -0.07) | 0.026 | -0.54 (-0.98, -0.10) | 0.015 | -0.60 (-1.12, -0.08) | 0.023 |
| **Beta (50)** | -1.24 (-1.75, -0.73) | <0.001 | -1.24 (-1.70, -0.77) | <0.01 | -1.32 (-1.82, -0.81) | <0.001 |
| **Gamma (110)** | -0.49 (-0.85, -0.14) | 0.007 | -0.49 (-0.82, -0.17) | 0.003 | -0.22 (-0.63, 0.20) | 0.306 |
| **Epsilon (37)** | -0.08 (-0.67, 0.51) | 0.786 | -0.08 (-0.62, 0.46) | 0.767 | -0.14 (-0.82, 0.54) | 0.683 |
| **Zeta (76)** | -0.21 (-0.63, 0.21) | 0.328 | -0.21 (-0.59, 0.18) | 0.287 | 0.11 (-0.38, 0.59) | 0.672 |
| **Iota (6)** | 0.03 (-1.41, 1.46) | 0.971 | 0.03 (-1.29, 1.34) | 0.968 | -0.46 (-2.37, 1.45) | 0.633 |
| **Delta (7)** | 0.28 (-1.05, 1.61) | 0.681 | 0.28 (-0.94, 1.50) | 0.654 | -0.28 (-1.81, 1.24) | 0.714 |
| **Lambda (59)** | -0.26 (-0.73, 0.22) | 0.288 | -0.26 (-0.69, 0.18) | 0.248 | -0.07 (-0.59, 0.45) | 0.792 |
| **Mu (53)** | -0.86 (-1.35, -0.36) | <0.001 | -0.86 (-1.31, -0.40) | <0.01 | -0.70 (-1.26, -0.15) | 0.013 |
| **Missing (344)** | -2.52 (-2.74, -2.29) | <0.001 | | | | |

© 2024 Fisher LH et al. *JAMA Network Open.*

**Multivariate model using Hamming distance**

As an exploratory analysis, we also used the Hamming distance of spike sequences from placebo infections to the Ancestral SARS-CoV-2 strain as an alternative to the variant.

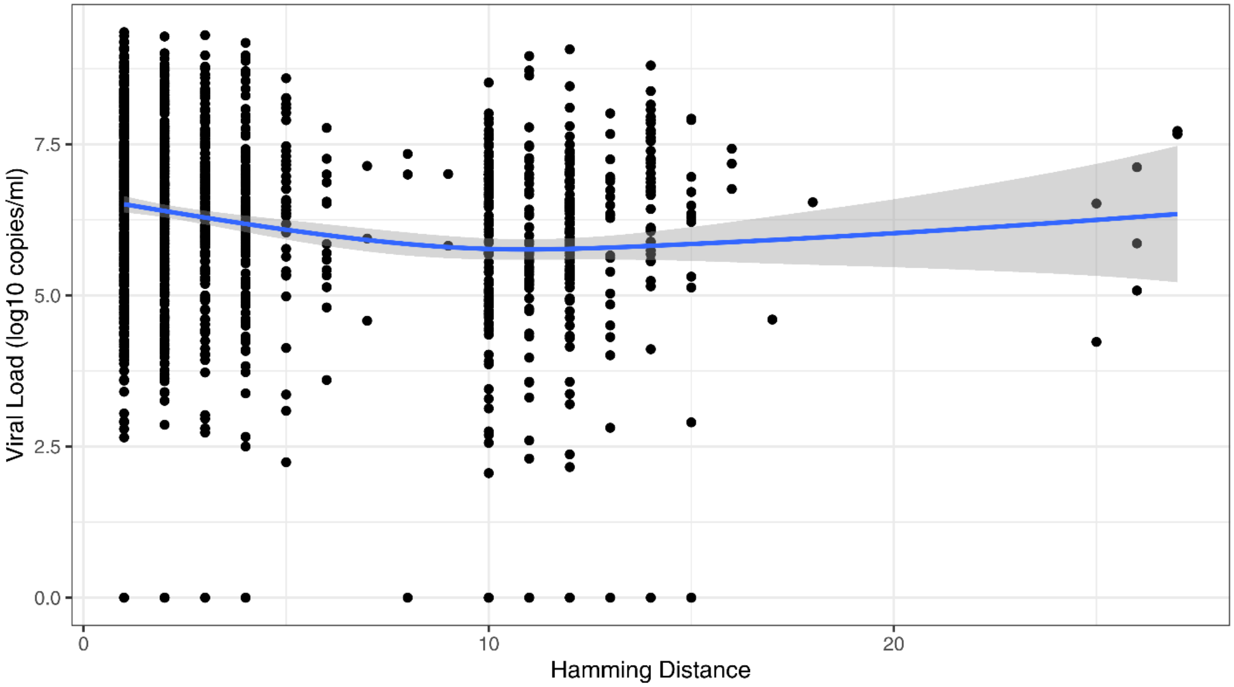


**eFigure 1. Scatter plot of viral load at diagnosis ($log_{10}$ copies/mL) by hamming distance of spike sequence to Ancestral SARS-CoV-2, for those placebo infections with successful sequencing.**

© 2024 Fisher LH et al. *JAMA Network Open.*

**eTable 4. Multivariate Linear Regression of $log_{10}$ viral load at diagnosis using Hamming distance.** N=1,323 infections with sequenced infections and with values for all covariates tested in multivariate model. Estimated mean difference in viral load at diagnosis ($log_{10}$ copies/mL) between each covariate category and the reference category, or per unit increase in the covariate in the case of continuous covariates, along with 95% confidence interval and nominal p-value, based on univariate linear regression models. Global nominal p-values from Wald Test to test coefficients for categorical variables with >2 levels. Adjusted p-values account for multiplicity and are corrected using the Holm method.

| Covariate | Mean Difference $log_{10}$(VL) (95% CI) | Nominal P-value | Global Nominal P-value | Adjusted P-value |
|---|---|---|---|---|
| Age Category (Ref: 18-29) | | | | |
| 30-39 | -0.08 (-0.38, 0.23) | 0.629 | 0.357 | 1.00 |
| 40-49 | 0.04 (-0.24, 0.32) | 0.76 | | |
| 50-64 | 0.14 (-0.13, 0.41) | 0.303 | | |
| 65+ | 0.24 (-0.11, 0.58) | 0.175 | | |
| Sex (Ref: Female) | | | | |
| Male | -0.00 (-0.18, 0.17) | 0.962 | | 1.00 |
| Race (Ref: White) | | | | |
| American Indian or Alaska Native | -0.16 (-0.63, 0.31) | 0.506 | 0.371 | 1.00 |
| Asian | -0.08 (-0.68, 0.53) | 0.799 | | |
| Black or African American | -0.37 (-0.75, 0.01) | 0.055 | | |
| Multiple | -0.21 (-0.66, 0.23) | 0.348 | | |
| Other | 0.56 (-0.41, 1.54) | 0.257 | | |
| Not Reported | -0.29 (-0.84, 0.25) | 0.296 | | |
| Ethnicity (Ref: Not Hispanic or Latino) | | | | |
| Hispanic or Latino | -0.05 (-0.34, 0.24) | 0.731 | 0.441 | 1.00 |
| Not Reported | 0.50 (-0.34, 1.34) | 0.24 | | |
| Infecting Variant | | | | |
| Hamming distance to Ancestral | -0.01 (-0.04, 0.01) | 0.315 | | 1.00 |
| Symptom Severity (Ref: Not Severe) | | | | |
| Severe | -0.22 (-0.47, 0.02) | 0.07 | | 0.628 |
| Days since COVID-19 Onset | -0.02 (-0.11, 0.06) | 0.579 | | 1.00 |
| COVID-19 Comorbidities (Ref: No) | | | | |
| Yes | 0.00 (-0.19, 0.20) | 0.974 | | 1 |
| Parent Protocol (Ref: Moderna) | | | | |
| AstraZeneca | -0.13 (-0.56, 0.29) | 0.532 | <0.001 | <0.001 |
| Janssen | -1.07 (-1.37, -0.78) | <0.001 | | |
| Novavax | -0.46 (-0.99, 0.08) | 0.098 | | |
| Country (Ref: USA) | | | | |
| Argentina | 0.20 (-0.28, 0.68) | 0.409 | 0.204 | 1.00 |
| Brazil | 0.40 (-0.00, 0.80) | 0.052 | | |
| Chile | 0.40 (-0.55, 1.35) | 0.413 | | |
| Colombia | 0.11 (-0.43, 0.66) | 0.68 | | |
| Mexico | -0.88 (-2.21, 0.45) | 0.194 | | |
| Peru | 0.51 (-0.11, 1.14) | 0.108 | | |
| South Africa | -0.05 (-0.62, 0.52) | 0.865 | | |

© 2024 Fisher LH et al. *JAMA Network Open.*

**Summary of complete case analysis (multivariate model among those with variant data, no imputation)**

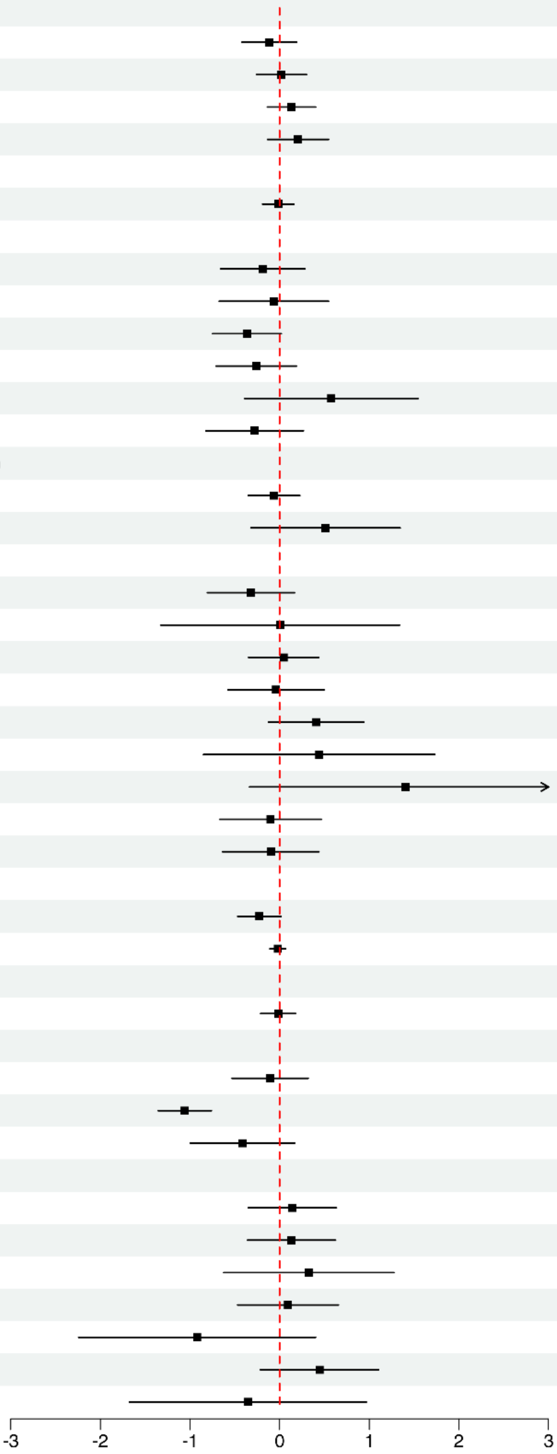


| Covariate | Mean Diff. in VL (95% CI) | Adjusted P |
|---|---|---|
| Age Category (Ref:18-29) | | |
| 30-39 | -0.11 (-0.42, 0.19) | 1.000 |
| 40-49 | 0.02 (-0.26, 0.30) | |
| 50-64 | 0.13 (-0.14, 0.40) | |
| 65+ | 0.21 (-0.13, 0.55) | |
| Sex (Ref:Female) | | |
| Male | -0.02 (-0.19, 0.16) | 1.000 |
| Race (Ref:White) | | |
| American Indian or Alaska Native | -0.19 (-0.66, 0.28) | 1.000 |
| Asian | -0.06 (-0.67, 0.55) | |
| Black or African American | -0.36 (-0.75, 0.02) | |
| Multiple | -0.26 (-0.71, 0.19) | |
| Other | 0.58 (-0.39, 1.55) | |
| Not Reported | -0.28 (-0.82, 0.27) | |
| Ethnicity (Ref:Not Hispanic or Latino) | | |
| Hispanic or Latino | -0.06 (-0.35, 0.23) | 1.000 |
| Not Reported | 0.51 (-0.32, 1.35) | |
| Infecting Variant (Ref:Ancestral) | | |
| Alpha | -0.32 (-0.81, 0.17) | 1.000 |
| Beta | 0.01 (-1.33, 1.34) | |
| Gamma | 0.05 (-0.35, 0.44) | |
| Epsilon | -0.04 (-0.58, 0.50) | |
| Zeta | 0.41 (-0.13, 0.94) | |
| Iota | 0.44 (-0.85, 1.73) | |
| Delta | 1.40 (-0.33, 3.14) | |
| Lambda | -0.10 (-0.67, 0.47) | |
| Mu | -0.10 (-0.64, 0.44) | |
| Symptom Severity (Ref:Not Severe) | | |
| Severe | -0.23 (-0.47, 0.02) | 0.610 |
| Days since COVID-19 Onset | -0.02 (-0.11, 0.07) | 1.000 |
| COVID-19 Co-Morbidities (Ref:No) | | |
| Yes | -0.02 (-0.21, 0.18) | 1.000 |
| Parent Protocol (Ref:Moderna) | | |
| AstraZeneca | -0.11 (-0.53, 0.32) | <0.001 |
| Janssen | -1.06 (-1.35, -0.76) | |
| Novavax | -0.41 (-0.99, 0.17) | |
| Country (Ref:USA) | | |
| Argentina | 0.14 (-0.35, 0.63) | 1.000 |
| Brazil | 0.13 (-0.36, 0.62) | |
| Chile | 0.33 (-0.62, 1.28) | |
| Colombia | 0.09 (-0.47, 0.66) | |
| Mexico | -0.92 (-2.24, 0.40) | |
| Peru | 0.45 (-0.21, 1.10) | |
| South Africa | -0.35 (-1.67, 0.97) | |

**eFigure 2. Estimated mean differences in SARS-CoV-2 viral load in nasal/NP swab at COVID-19 diagnosis, among those with successful variant calls (N = 1,323; adjusted $R^2$ = 0.106).** Forest plot illustrating estimated mean difference in $log_{10}$ copies/mL SARS-CoV-2 viral load between groups defined by participant or COVID-19 disease characteristics, based on multivariate regression analysis. 95% confidence intervals and Holm-adjusted p-values are provided. Days since Covid-19 onset is defined as the number of calendar days between protocol-defined onset of COVID-19 and the specimen collection corresponding to diagnosis.

© 2024 Fisher LH et al. *JAMA Network Open.*

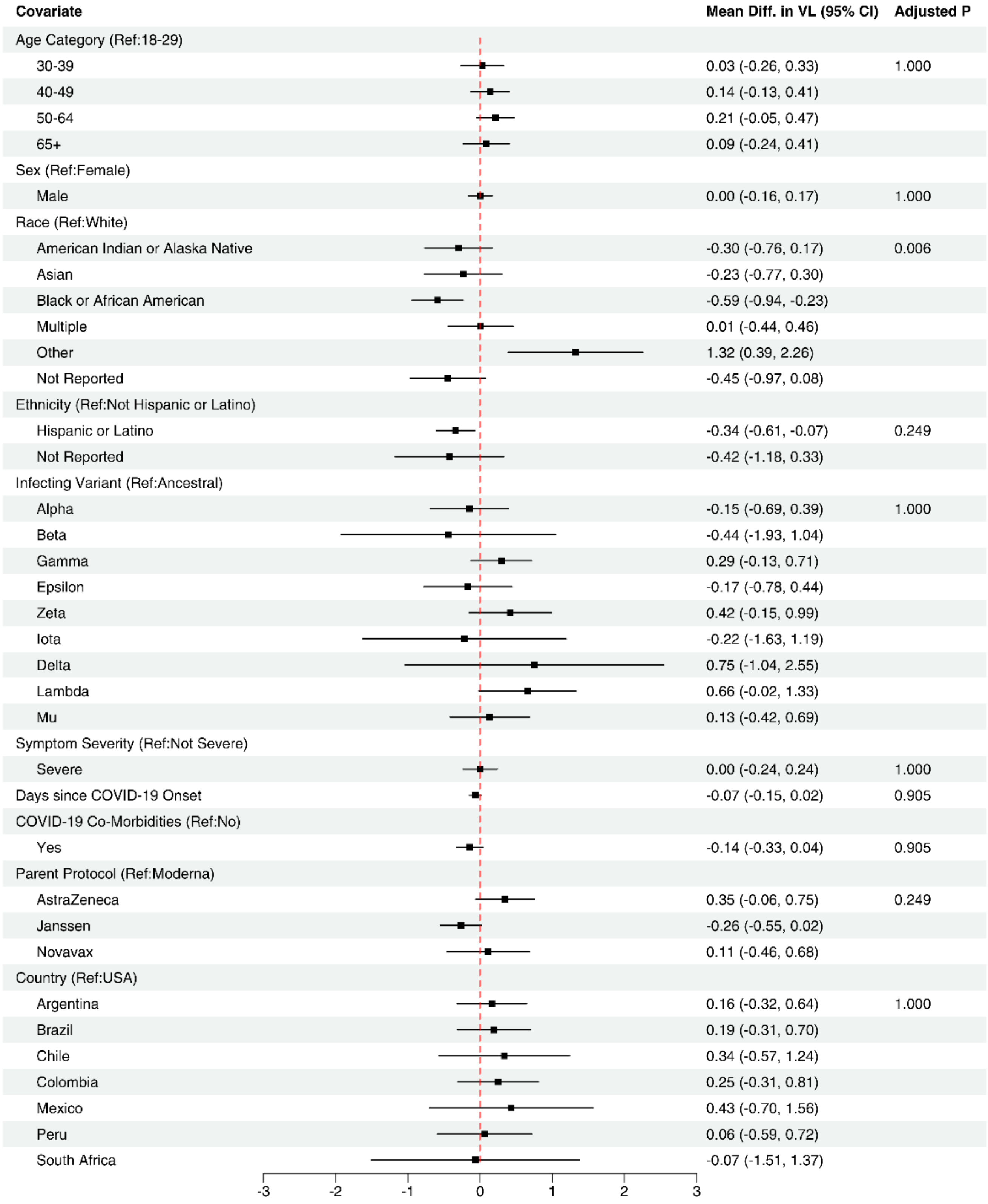


**eFigure 3. Estimated mean differences in SARS-CoV-2 viral load in swabs at COVID-19 diagnosis on the subset of participants with quantifiable viral load at diagnosis, imputing missing variants (N=1,599; adjusted $R^2$ = 0.044).** Forest plot illustrating estimated mean difference in $\log_{10}$ copies/mL SARS-CoV-2 viral load between groups defined by participant or COVID-19 disease characteristics, based on multivariate regression analysis with imputed variants. 95% confidence intervals and Holm-adjusted p-values are provided.

© 2024 Fisher LH et al. *JAMA Network Open.*

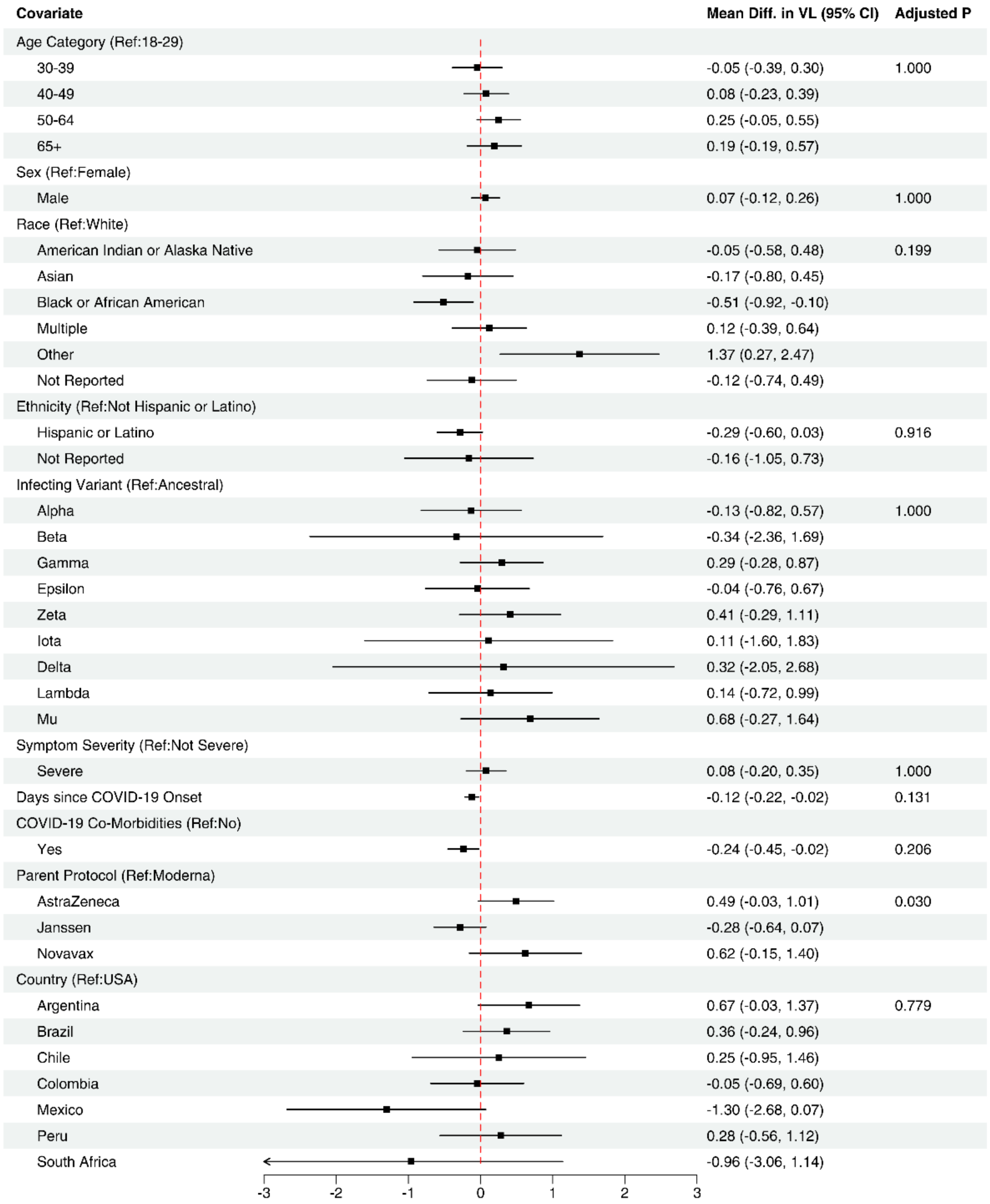


**eFigure 4a. Summary of parametric results from GAM analysis with country-specific temporal regression splines (N = 1,667; adjusted $R^2$ = 0.081).** Estimated mean difference in $\log_{10}$ viral load (VL) at diagnosis between each covariate category and the reference category, or per unit increase in the covariate in the case of continuous covariates, along with 95% confidence interval and nominal p-value, based on univariate linear regression models. Median global nominal p-values from Wald Test to test coefficients for categorical variables with >2 levels. Adjusted p-values account for multiplicity and are corrected using the Holm method. Days since Covid-19 onset is defined as the number of calendar days between protocol-defined onset of COVID-19 and the specimen collection corresponding to diagnosis.

© 2024 Fisher LH et al. *JAMA Network Open.*

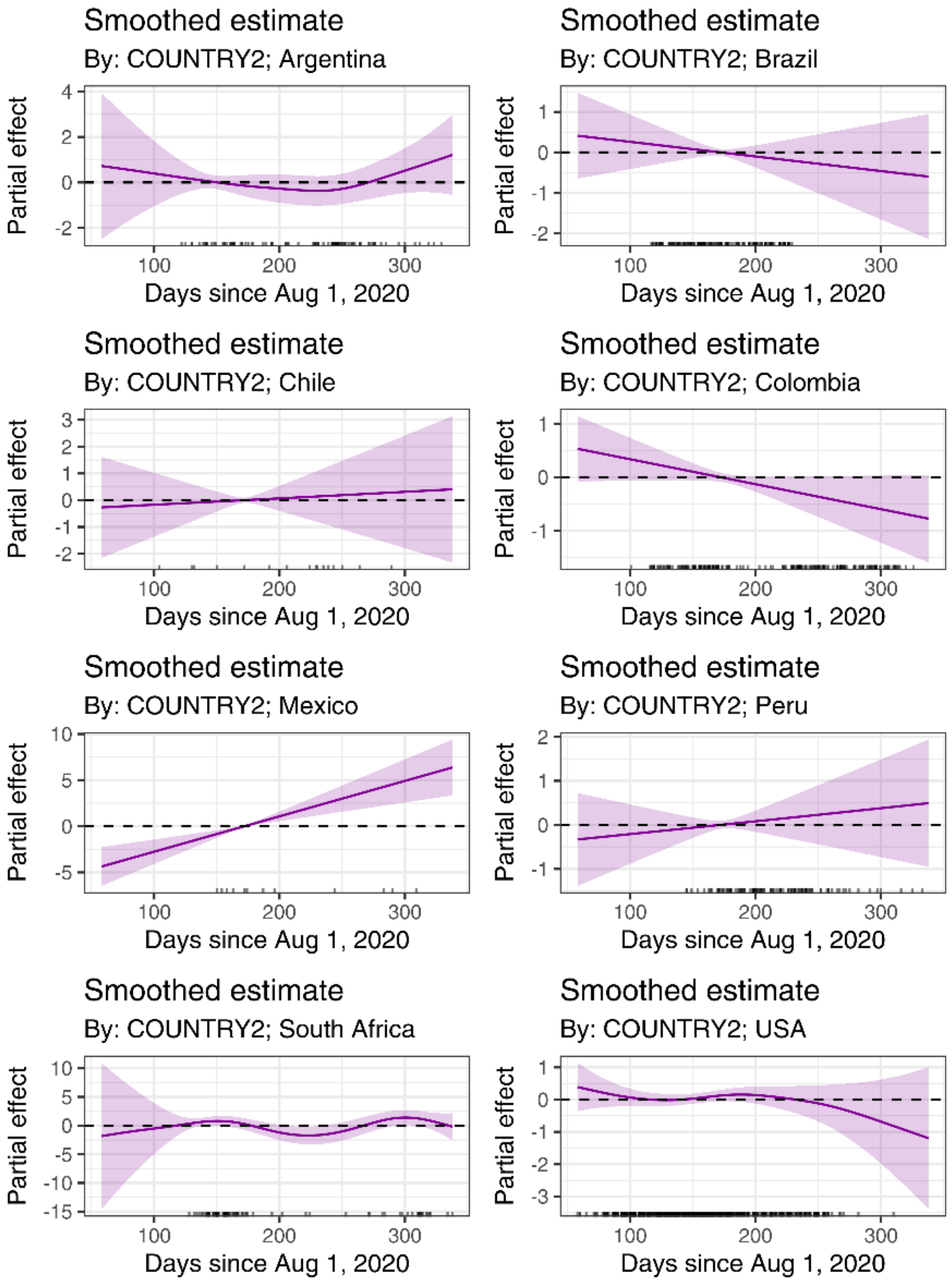


**eFigure 4b. Estimated country-level temporal smoothers.** Ticks at bottom of each panel indicate contributing cases.

© 2024 Fisher LH et al. *JAMA Network Open.*

## Subgroup Sensitivity Analyses

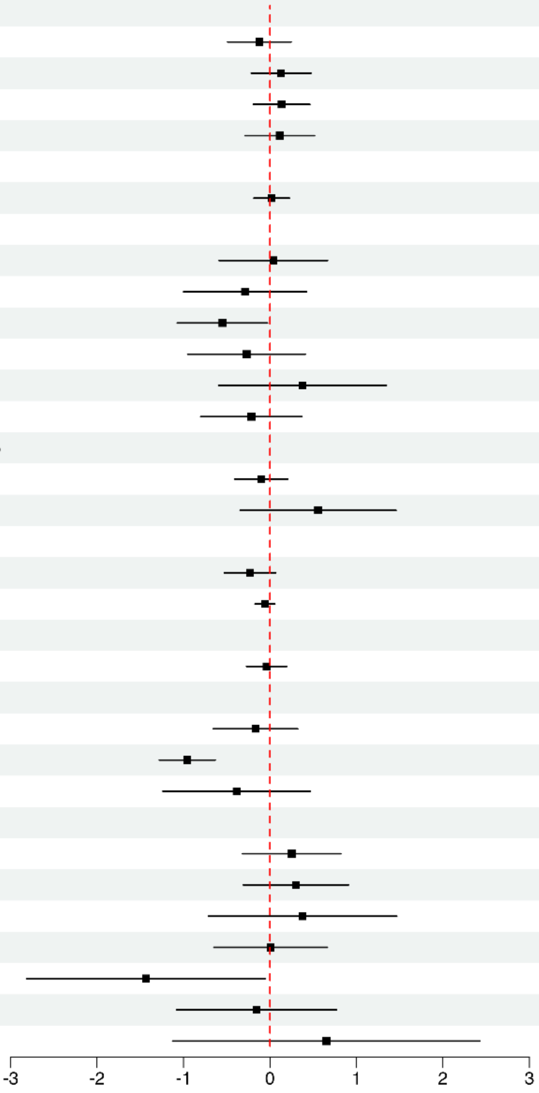


| Covariate | Mean Diff. in VL (95% CI) | Nominal P | Wald P | Adjusted P |
|---|---|---|---|---|
| Age Category (Ref:18-29) | | | | |
| 30-39 | -0.12 (-0.49, 0.25) | 0.515 | 0.501 | 1.000 |
| 40-49 | 0.13 (-0.22, 0.47) | 0.464 | | |
| 50-64 | 0.13 (-0.19, 0.46) | 0.418 | | |
| 65+ | 0.11 (-0.29, 0.52) | 0.580 | | |
| Sex (Ref:Female) | | | | |
| Male | 0.02 (-0.19, 0.22) | 0.853 | | 1.000 |
| Race (Ref:White) | | | | |
| American Indian or Alaska Native | 0.04 (-0.59, 0.67) | 0.902 | 0.352 | 1.000 |
| Asian | -0.29 (-1.00, 0.42) | 0.427 | | |
| Black or African American | -0.55 (-1.07, -0.03) | 0.039 | | |
| Multiple | -0.27 (-0.95, 0.41) | 0.434 | | |
| Other | 0.38 (-0.59, 1.35) | 0.446 | | |
| Not Reported | -0.21 (-0.80, 0.37) | 0.472 | | |
| Ethnicity (Ref:Not Hispanic or Latino) | | | | |
| Hispanic or Latino | -0.10 (-0.41, 0.20) | 0.518 | 0.344 | 1.000 |
| Not Reported | 0.56 (-0.34, 1.46) | 0.226 | | |
| Symptom Severity (Ref:Not Severe) | | | | |
| Severe | -0.23 (-0.53, 0.07) | 0.130 | | 1.000 |
| Days since COVID-19 Onset | -0.06 (-0.17, 0.06) | 0.324 | | 1.000 |
| COVID-19 Co-Morbidities (Ref:No) | | | | |
| Yes | -0.04 (-0.27, 0.19) | 0.747 | | 1.000 |
| Parent Protocol (Ref:Moderna) | | | | |
| AstraZeneca | -0.17 (-0.66, 0.32) | 0.502 | <0.001 | <0.001 |
| Janssen | -0.96 (-1.28, -0.63) | <0.001 | | |
| Novavax | -0.39 (-1.24, 0.47) | 0.374 | | |
| Country (Ref:USA) | | | | |
| Argentina | 0.25 (-0.32, 0.82) | 0.385 | 0.397 | 1.000 |
| Brazil | 0.30 (-0.31, 0.91) | 0.332 | | |
| Chile | 0.38 (-0.71, 1.47) | 0.496 | | |
| Colombia | 0.01 (-0.65, 0.66) | 0.981 | | |
| Mexico | -1.43 (-2.81, -0.05) | 0.042 | | |
| Peru | -0.16 (-1.08, 0.77) | 0.742 | | |
| South Africa | 0.65 (-1.12, 2.43) | 0.471 | | |

**eFigure 5. Multivariate linear regression of $\log_{10}$ viral load at diagnosis on the subset of participants infected with the Ancestral variant Only (N = 867; adjusted $R^2$ = 0.104).** Estimated mean difference in viral load at diagnosis ($\log_{10}$ copies/mL) between each covariate category and the reference category, or per unit increase in the covariate in the case of continuous covariates, along with 95% confidence interval and nominal p-value, based on univariate linear regression models. Global nominal p-values from Wald Test to test coefficients for categorical variables with >2 levels. Adjusted p-values account for multiplicity and are corrected using the Holm method.

© 2024 Fisher LH et al. *JAMA Network Open.*

| Covariate | Mean Diff. in VL (95% CI) | Nominal P | Global P | Adjusted P |
|---|---|---|---|---|
| Age Category (Ref:18-29) | | | | |
| 30-39 | -0.06 (-0.53, 0.41) | 0.798 | 0.860 | 0.869 |
| 40-49 | 0.11 (-0.33, 0.54) | 0.621 | | |
| 50-64 | 0.13 (-0.28, 0.54) | 0.537 | | |
| 65+ | 0.02 (-0.47, 0.51) | 0.934 | | |
| Sex (Ref:Female) | | | | |
| Male | 0.15 (-0.11, 0.40) | 0.259 | | 0.835 |
| Race (Ref:White) | | | | |
| American Indian or Alaska Native | -0.68 (-2.15, 0.80) | 0.369 | 0.013 | 0.114 |
| Asian | -0.18 (-0.83, 0.48) | 0.596 | | |
| Black or African American | -0.76 (-1.29, -0.24) | 0.004 | | |
| Multiple | -0.66 (-1.86, 0.54) | 0.279 | | |
| Other | 1.33 (0.21, 2.45) | 0.020 | | |
| Not Reported | 0.16 (-0.68, 1.00) | 0.715 | | |
| Ethnicity (Ref:Not Hispanic or Latino) | | | | |
| Hispanic or Latino | -0.43 (-0.78, -0.08) | 0.016 | 0.053 | 0.371 |
| Not Reported | -0.03 (-1.12, 1.06) | 0.955 | | |
| Infecting Variant (Ref:Ancestral) | | | | |
| Alpha | -0.28 (-1.13, 0.57) | 0.510 | 0.209 | 0.835 |
| Beta | -2.24 (-6.40, 1.93) | 0.291 | | |
| Gamma | 0.08 (-2.70, 2.87) | 0.954 | | |
| Epsilon | -0.16 (-0.86, 0.55) | 0.664 | | |
| Zeta | 1.19 (-1.25, 3.63) | 0.336 | | |
| Iota | -0.25 (-2.13, 1.63) | 0.792 | | |
| Delta | -4.36 (-10.44, 1.72) | 0.147 | | |
| Symptom Severity (Ref:Not Severe) | | | | |
| Severe | 0.16 (-0.24, 0.55) | 0.435 | | 0.869 |
| Days since COVID-19 Onset | -0.18 (-0.33, -0.03) | 0.020 | | 0.163 |
| COVID-19 Co-Morbidities (Ref:No) | | | | |
| Yes | -0.27 (-0.55, 0.01) | 0.060 | | 0.371 |
| Parent Protocol (Ref:Moderna) | | | | |
| AstraZeneca | 0.06 (-0.48, 0.61) | 0.819 | 0.101 | 0.507 |
| Janssen | -0.42 (-0.81, -0.03) | 0.036 | | |
| Novavax | 0.08 (-0.70, 0.85) | 0.847 | | |

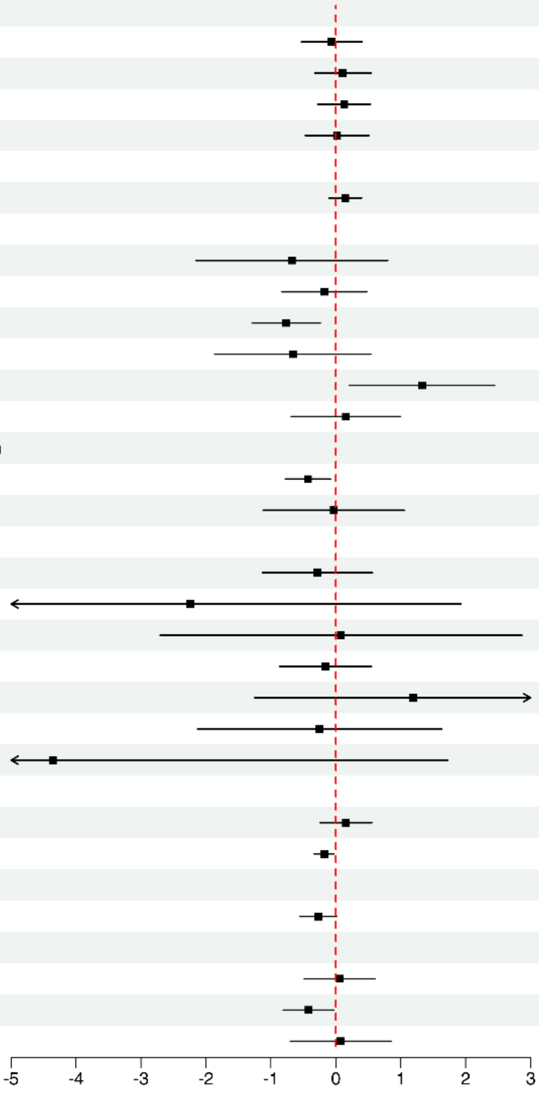


**eFigure 6. Estimated mean differences in SARS-CoV-2 viral load in swabs at COVID-19 diagnosis on the subset of participants living in the US, imputing missing variants (N=995; adjusted $R^2$ = 0.047).** Forest plot illustrating estimated mean difference in $log_{10}$ copies/mL SARS-CoV-2 viral load between groups defined by participant or COVID-19 disease characteristics, based on multivariate regression analysis with imputed variants. 95% confidence intervals and Holm-adjusted p-values are provided. Median p-values over multiple imputations are reported.

© 2024 Fisher LH et al. *JAMA Network Open.*

| Covariate | Mean Diff. in VL (95% CI) | Nominal P | Wald P | Adjusted P |
|---|---|---|---|---|
| Age Category (Ref:18-29) | | | | |
| 30-39 | 0.09 (-0.38, 0.57) | 0.698 | 0.508 | 1.000 |
| 40-49 | 0.15 (-0.26, 0.55) | 0.478 | | |
| 50-64 | 0.32 (-0.08, 0.71) | 0.118 | | |
| 65+ | 0.35 (-0.16, 0.86) | 0.173 | | |
| Sex (Ref:Female) | | | | |
| Male | -0.07 (-0.34, 0.19) | 0.578 | | 1.000 |
| Race (Ref:White) | | | | |
| American Indian or Alaska Native | 0.06 (-0.54, 0.65) | 0.853 | 0.611 | 1.000 |
| Asian | -0.71 (-1.81, 0.39) | 0.206 | | |
| Black or African American | -0.24 (-0.78, 0.30) | 0.386 | | |
| Multiple | 0.28 (-0.30, 0.86) | 0.344 | | |
| Not Reported | 0.03 (-0.70, 0.77) | 0.926 | | |
| Ethnicity (Ref:Not Hispanic or Latino) | | | | |
| Hispanic or Latino | -0.29 (-0.84, 0.26) | 0.297 | 0.574 | 1.000 |
| Not Reported | -0.16 (-1.15, 0.83) | 0.745 | | |
| Infecting Variant (Ref:Ancestral) | | | | |
| Alpha | -0.55 (-1.33, 0.22) | 0.163 | 0.804 | 1.000 |
| Beta | 0.07 (-1.64, 1.79) | 0.933 | | |
| Gamma | 0.08 (-0.44, 0.61) | 0.753 | | |
| Epsilon | 0.08 (-0.96, 1.12) | 0.881 | | |
| Zeta | 0.34 (-0.40, 1.08) | 0.368 | | |
| Iota | -0.01 (-1.94, 1.92) | 0.991 | | |
| Delta | 1.09 (-1.08, 3.26) | 0.324 | | |
| Lambda | -0.12 (-0.88, 0.64) | 0.762 | | |
| Mu | 0.07 (-0.65, 0.79) | 0.849 | | |
| Symptom Severity (Ref:Not Severe) | | | | |
| Severe | -0.06 (-0.41, 0.29) | 0.743 | | 1.000 |
| Days since COVID-19 Onset | -0.14 (-0.24, -0.04) | 0.008 | | 0.070 |
| COVID-19 Co-Morbidities (Ref:No) | | | | |
| Yes | -0.16 (-0.44, 0.12) | 0.269 | | 1.000 |
| Country (Ref:USA) | | | | |
| Argentina | 0.42 (-0.25, 1.10) | 0.217 | 0.164 | 1.000 |
| Brazil | 0.36 (-0.32, 1.04) | 0.301 | | |
| Chile | 0.43 (-0.96, 1.83) | 0.543 | | |
| Colombia | -0.21 (-0.99, 0.57) | 0.593 | | |
| Mexico | -1.57 (-3.06, -0.08) | 0.039 | | |
| Peru | 0.17 (-0.70, 1.05) | 0.696 | | |
| South Africa | -0.86 (-2.54, 0.81) | 0.311 | | |

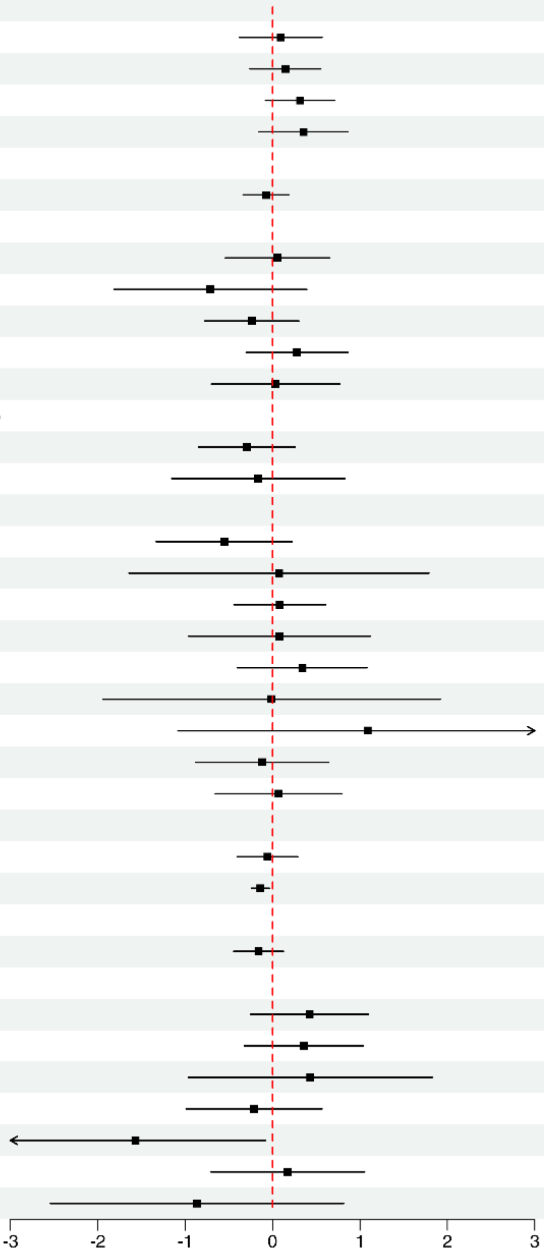


**eFigure 7. Estimated mean differences in SARS-CoV-2 viral load in swabs at COVID-19 diagnosis on the subset of placebo participants enrolled in the Janssen trial, imputing missing variants (N=916; adjusted $R^2$ = 0.025).** Forest plot illustrating estimated mean difference in $\log_{10}$ copies/mL SARS-CoV-2 viral load between groups defined by participant or COVID-19 disease characteristics, based on multivariate regression analysis with imputed variants. 95% confidence intervals and Holm-adjusted p-values are provided.

© 2024 Fisher LH et al. *JAMA Network Open.*

## Predictors of Severe COVID-19

Viral load measurements alone were found to be poor predictors of severe COVID-19. Viral load at diagnosis had a CV-AUC of 0.52 (95% CI: 0.47 to 0.57) and the area under the viral load curve (VL-AUC) had a CV-AUC of 0.49 (95% CI: 0.42 to 0.57). The predictive performance of the ensemble model, discrete Superlearner, and individual learners are summarized in **eFigure 8**.

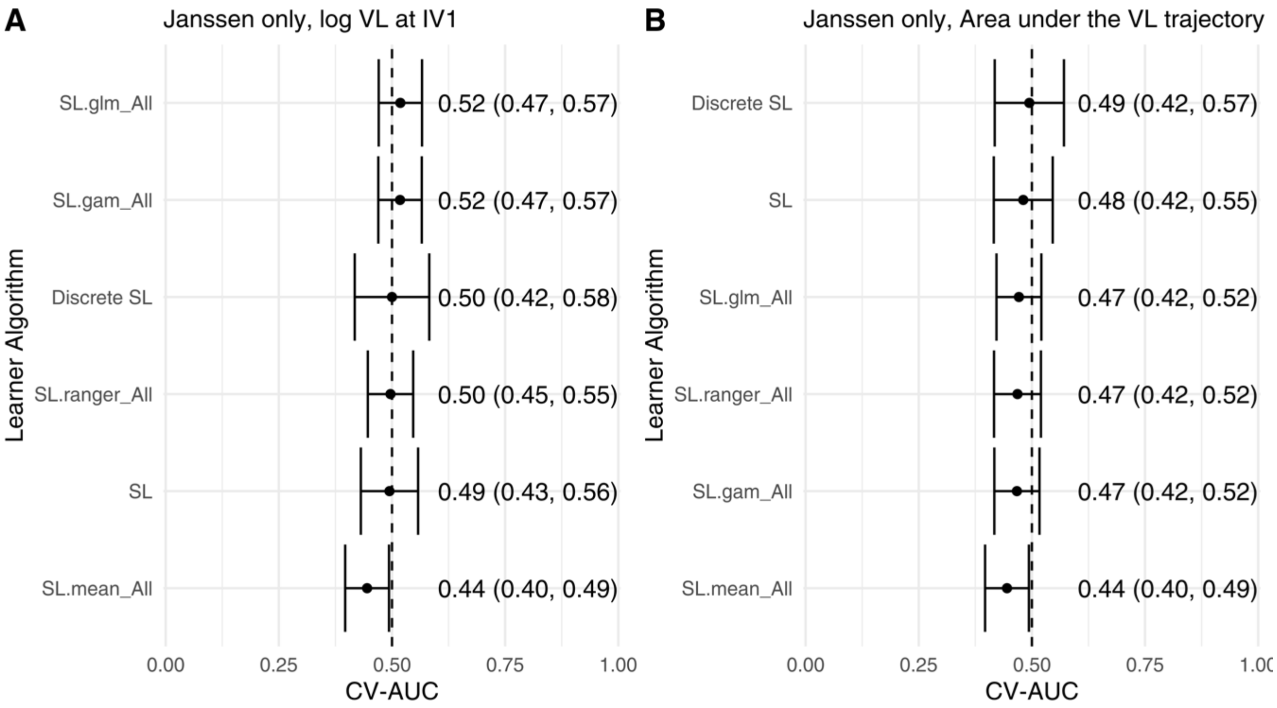


**eFigure 8. Summary of the utility of log viral load at diagnosis (A) and the area under the VL trajectory curve (B) in predicting severe COVID-19.** For each analysis, we summarize the cross-validated area under the ROC curve estimates and 95% confidence intervals for each of the candidate algorithms, in additional to the discrete and continuous SuperLearner.

In **eFigure 9**, the estimated marginal feature importance for all available covariates was plotted in descending order and neither viral load measurement broke the top 10 most important variables in predicting severe COVID-19. Participant characteristics, including age, race, country, and various comorbidities were ranked as higher importance than both viral load measurements, which is consistent with the literature;[14] variant was among the top-ranked variables marginally, although none of these features were found to be statistically significant.

© 2024 Fisher LH et al. *JAMA Network Open.*

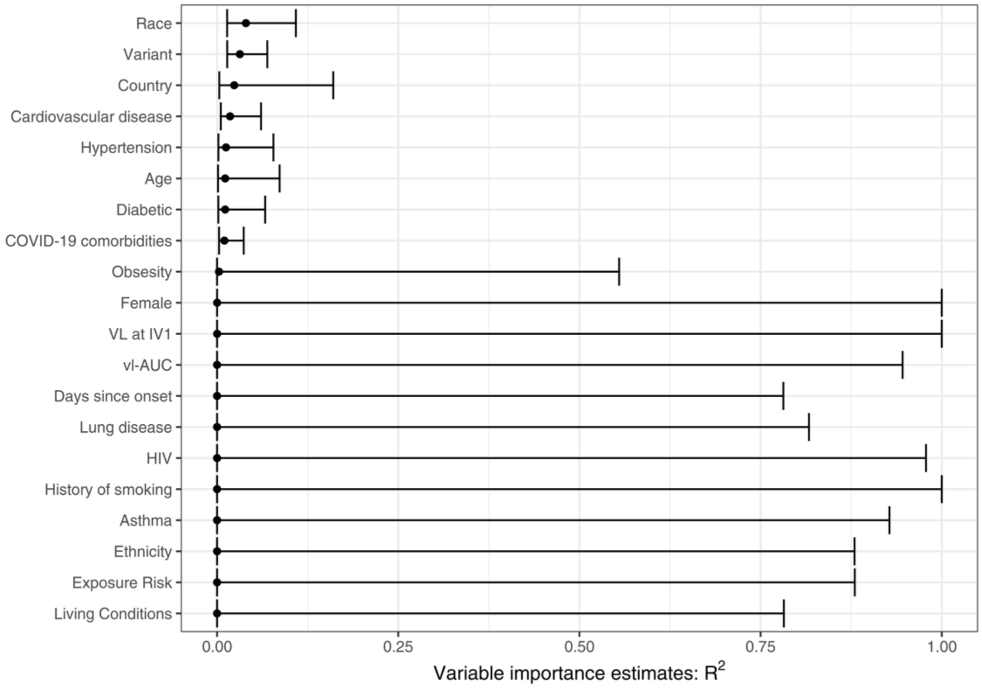


**eFigure 9. Marginal variable importance measures for all available baseline characteristics and covariates collected around the time of infection.**

Prediction of severe COVID-19 disease was improved with the inclusion of additional baseline characteristics, with a CV-AUC of 0.71 (95% CI: 0.67 to 0.75; **eFigure 10**).

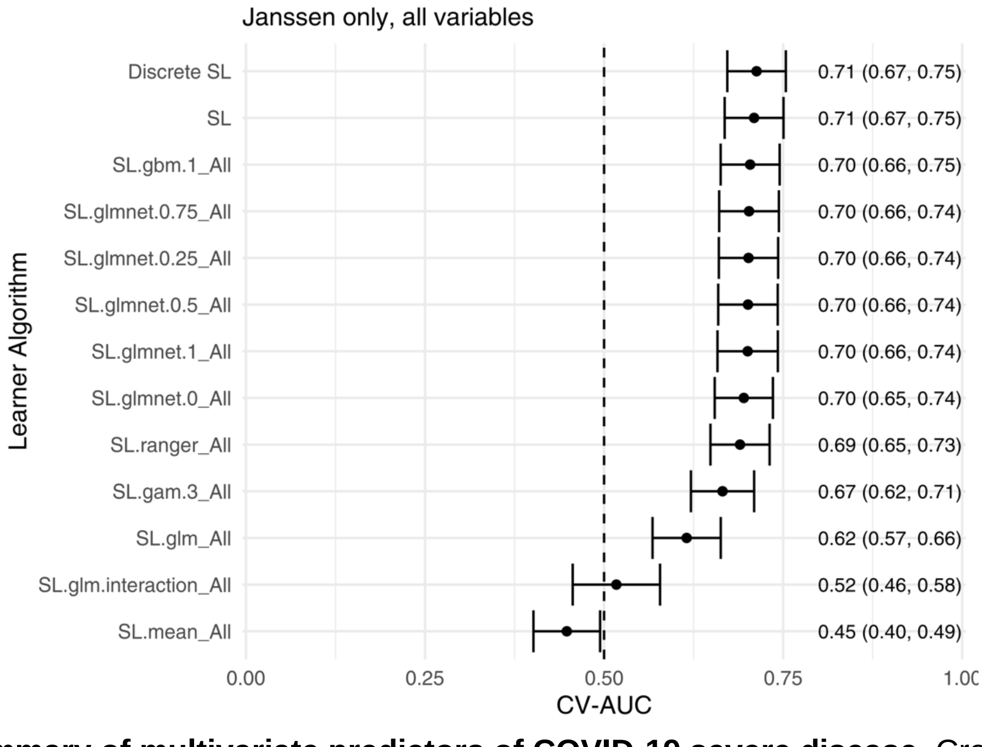


**eFigure 10**. **Summary of multivariate predictors of COVID-19 severe disease.** Cross-validated area under the ROC curve estimates and 95% confidence intervals for each of the candidate algorithms, in additional to the discrete and continuous SuperLearner.

When adjusting for other baseline and infection characteristics, log viral load at diagnosis (IV1) had the highest-ranked conditional variable importance measure (**eFigure 11**). This suggests that after accounting for other known predictors, log viral load at diagnosis

© 2024 Fisher LH et al. *JAMA Network Open.*

(IV1) does improve the prediction of severe COVID-19. However, the wide 95% confidence intervals suggest that the improvements in prediction with log viral load measurements are modest at best.

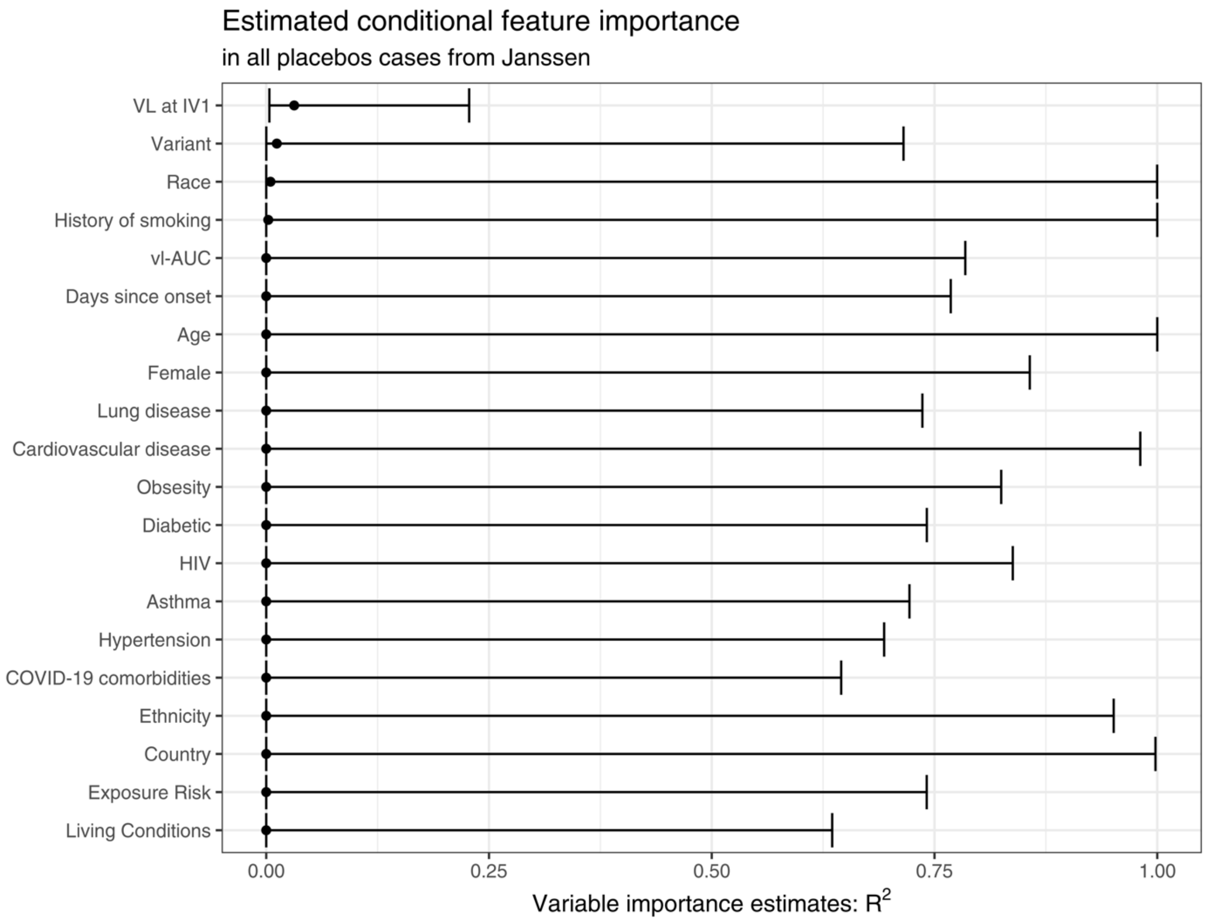


**eFigure 11. Estimated conditional variable importance measures and 95% CI for the features in the prediction of severe COVID-19.**

# eReferences


1. Theodore DA, Branche AR, Zhang L, et al. Clinical and Demographic Factors Associated With COVID-19, Severe COVID-19, and SARS-CoV-2 Infection in Adults: A Secondary Cross-Protocol Analysis of 4 Randomized Clinical Trials. *JAMA Netw Open*. Jul 3 2023;6(7):e2323349. doi:10.1001/jamanetworkopen.2023.23349
2. United States Food and Drug Administration. Emergency Use Authorization (EUA) Summary COVID-19 RT-PCR Test (Laboratory Corporation of America) https://www.fda.gov/media/136151/download
3. Pajon R, Paila YD, Girard B, et al. Initial analysis of viral dynamics and circulating viral variants during the mRNA-1273 Phase 3 COVE trial. *Nat Med*. Apr 2022;28(4):823-830. doi:10.1038/s41591-022-01679-5
4. Abbott. Abbott RealTime SARS-CoV-2 Instructions for Use. Accessed September 1, 2023. https://www.fda.gov/media/136258/download
5. Chang W, Cheng J, Allaire J, et al. shiny: Web Application Framework for R. R package version 1.7.4.1. https://shiny.posit.co/
6. Khare S, Gurry C, Freitas L, et al. GISAID's Role in Pandemic Response. *China CDC Wkly*. Dec 3 2021;3(49):1049-1051. doi:10.46234/ccdcw2021.255
7. Katoh K, Standley DM. MAFFT multiple sequence alignment software version 7: improvements in performance and usability. *Mol Biol Evol*. Apr 2013;30(4):772-80. doi:10.1093/molbev/mst010
8. COVID-19 Viral Genome Analysis Pipeline. Accessed September, 2021. https://cov.lanl.gov/components/sequence/COV/annt/curated_variants.comp
9. Chen T, Guestrin C. XGBoost: A Scalable Tree Boosting System. presented at: Proceedings of the 22nd ACM SIGKDD International Conference on Knowledge Discovery and Data Mining; 2016; San Francisco, California, USA. https://doi.org/10.1145/2939672.2939785
10. van Buuren S, Groothuis-Oudshoorn K. mice: Multivariate Imputation by Chained Equations in R. *Journal of Statistical Software*. 12/12 2011;45(3):1 - 67. doi:10.18637/jss.v045.i03
11. Bolt MA, MaWhinney S, Pattee JW, Erlandson KM, Badesch DB, Peterson RA. Inference following multiple imputation for generalized additive models: an investigation of the median p-value rule with applications to the Pulmonary Hypertension Association Registry and Colorado COVID-19 hospitalization data. *BMC Med Res Methodol*. May 21 2022;22(1):148. doi:10.1186/s12874-022-01613-w
12. Polley EC, van der Laan MJ. Super Learner In Prediction. *UC Berkeley Division of Biostatistics Working Paper Series*. 2010;Working Paper 266doi:https://biostats.bepress.com/ucbbiostat/paper266
13. Williamson BD, Gilbert PB, Simon NR, Carone M. A General Framework for Inference on Algorithm-Agnostic Variable Importance. *Journal of the American Statistical Association*. 2021:1-14. doi:10.1080/01621459.2021.2003200
14. Salto-Alejandre S, Berastegui-Cabrera J, Camacho-Martinez P, et al. SARS-CoV-2 viral load in nasopharyngeal swabs is not an independent predictor of unfavorable outcome. *Sci Rep*. Jun 21 2021;11(1):12931. doi:10.1038/s41598-021-92400-y


© 2024 Fisher LH et al. *JAMA Network Open.*
