# Supplementary material for: SARS-CoV-2 Viral Load in the Nasopharynx at Time of First Infection Among Unvaccinated Individuals: A Secondary Cross-Protocol Analysis of 4 Randomized Trials
Source: JAMA Netw Open. 2024 May 23;7(5):e2412835. doi: 10.1001/jamanetworkopen.2024.12835 (PMC11117088; doi:10.1001/jamanetworkopen.2024.12835)
Supplement: Supplement 3. — Data Sharing Statement [file jamanetwopen-e2412835-s003.pdf]

Data Sharing Statement

Fisher. SARS-CoV-2 Viral Load in the Nasopharynx at Time of First Infection Among Unvaccinated Individuals. *JAMA Netw Open*. Published May 23, 2024. doi:10.1001/jamanetworkopen.2024.12835

Data
Data available: No

Additional Information
Explanation for why data not available: The data availability will be at the discretion of the original study sponsors.
